# Coactivator Associated Arginine Methyltransferase 1 Modulates Cartilage Degeneration and Chondrocyte Apoptosis in Osteoarthritis by Regulating ERK1/2 Signaling Pathway

**DOI:** 10.1111/acel.70122

**Published:** 2025-07-03

**Authors:** Jie Yuan, Jingyuan Tian, Ruxing Liu, Xiaoting Qiu, Dongqin He, Guanghui He, Tao Zhang, Pengcui Li, Bin Zhao, Yongfeng Wang

**Affiliations:** ^1^ Department of Orthopedics Second Hospital of Shanxi Medical University Taiyuan China; ^2^ Department of Shanxi Key Laboratory of Bone and Soft Tissue Injury Repair Second Hospital of Shanxi Medical University Taiyuan China; ^3^ Clinical Laboratory of the First People's Hospital of Zhaotong Zhaotong China

**Keywords:** apoptosis, CARM1, cartilage degeneration, ERK1/2, osteoarthritis

## Abstract

This study investigates the role and mechanism of Coactivator Associated Arginine Methyltransferase 1 (CARM1) in osteoarthritis (OA). OA is a prevalent joint disease characterized by cartilage degradation, subchondral bone remodeling, and inflammation. Our research revealed that CARM1 expression is significantly increased in the cartilage tissues of OA patients and OA model mice. Experimental results showed that inhibiting CARM1 reduces cartilage matrix degradation and chondrocyte apoptosis, while overexpression of CARM1 exacerbates these conditions. Mechanistically, CARM1 regulates OA progression through the phosphorylation of the ERK1/2 signaling pathway. Inhibition of CARM1 suppresses ERK1/2 activation, thereby reducing extracellular matrix degradation and chondrocyte apoptosis. These findings suggest that the CARM1‐ERK1/2 axis is crucial in modulating cartilage matrix metabolism and chondrocyte apoptosis in OA, highlighting CARM1 as a potential therapeutic target for OA treatment.

## Graphical abstract

The authors' research shows that CARM1 regulates cartilage degeneration in osteoarthritis through the ERK1/2 signaling pathway, suggesting its potential as a therapeutic target and providing valuable insights into the mechanisms of cartilage degradation and inflammation in the disease.

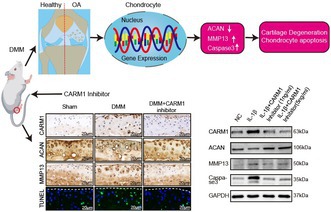

## 1 Introduction

Osteoarthritis (OA) is a progressive joint disease influenced by factors such as gender, age, obesity, joint injury, and genetics (Charlier et al. 2019; Lin et al. 2021). Affecting an estimated 500 million people globally, OA imposes a significant economic burden (Hunter et al. 2020). Its main manifestations include cartilage degradation, subchondral bone remodeling, synovial inflammation, and osteophyte formation (Guan et al. 2018), driven by disrupted cartilage matrix metabolism (Glyn‐Jones et al. 2015). The pathogenesis of OA involves decreased anabolism and proliferation, increased catabolism, and elevated apoptosis (Liu et al. 2019; Kim, Jeon, et al. 2014). Despite its prevalence, there are no definitive treatments for OA, highlighting the need to understand its underlying mechanisms and develop effective therapies.

Members of the protein arginine methyltransferase (PRMT) protein family, including PRMT1 (Xia et al. 2018) and PRMT5 (Dong et al. 2020; Chen, Zeng, et al. 2017), have been reported to be associated with OA. Coactivator Associated Arginine Methyltransferase 1 (CARM1), also known as protein arginine methyltransferase 4 (PRMT4), is an important member of the protein arginine methyltransferases (PRMTs) family (Teyssier et al. 2002). Prior research has demonstrated the pivotal involvement of CARM1 in the modulation of diverse cellular processes, including RNA processing, transcription activation, tumorigenesis, cancer progression, cell growth, differentiation, and apoptosis (Sanchez et al. 2016). Significantly, alterations in CARM1 expression, primarily characterized by up‐regulation, have been consistently documented in numerous human cancer types (Bertozzi et al. 2021; Grypari et al. 2021; Lu et al. 2020). Recently, a study has shown that the expressions of ERK1/2, PARS40, and GSK3β are affected by CARM1, and the over‐expression of CARM1 promotes the proliferation of human osteosarcoma cells through the *p‐*GSK3β signaling pathway (Li et al. 2017). Besides, it has also been shown that CARM1 can act as a coactivator of transcription factors other than nuclear receptors, such as NF‐kB, P53, and Cyclin E1 (Miao et al. 2006; An et al. 2004). However, the biological role of CARM1 in OA remains unclear and has not been studied yet.

ERK1/2 is a protein kinase that operates within the Ras–Raf–MEK–ERK signaling pathway, also recognized as the MAPK pathway (Wortzel and Seger 2011). This cascade is involved in regulating multiple processes, including cell cycle progression, cell adhesion, cell survival, cell migration, differentiation, transcription, proliferation, and metabolism (Roskoski Jr. 2012). ERK1/2 can be activated by cytokines in OA (Latourte et al. 2017), which is related to osteophyte formation (He et al. 2020) and cartilage calcification (Yue et al. 2019) and is mostly involved in regulating cell proliferation and apoptosis, and cartilage matrix metabolism (Sun et al. 2015; Kumar et al. 2021; Khan et al. 2017). During OA, the activation of the ERK1/2 signaling pathway inhibits chondrocyte anabolism and promotes chondrocyte catabolism and apoptosis (Xu et al. 2021; Prasadam et al. 2012). Since our preliminary experiment suggested CARM1 could interact with ERK1/2, we speculated that CARM1 may function by targeting ERK1/2.

This study focused on assessing the impact and mechanisms of CARM1 in OA degeneration. We demonstrated CARM1 accumulation in articular cartilage and IL‐1β‐treated chondrocytes in aging C57BL/6 mice and patients with knee OA. In vitro and in vivo inhibition of CARM1 reduced OA‐related cartilage degeneration, while its overexpression stimulated it. We found that the CARM1‐ERK1/2 axis regulates OA progression by affecting anabolism, catabolism, and apoptosis. This pioneering research highlights the biological significance of CARM1 in OA, suggesting its potential as a novel therapeutic target for OA treatment.

## 2 Materials and Methods

### 2.1 Clinical Tissue Samples

Specimens of knee cartilage from healthy people undergoing traumatic amputation (*n* = 6) and OA cartilage specimens from OA patients undergoing total knee replacement (*n* = 6). All patients with OA are diagnosed by symptoms, signs, imaging, and laboratory tests.

### 2.2 Animal Model

To simulate OA, we used two mouse models: the aging model and the destabilization of the medial meniscus (DMM) model, both with male C57BL/6 mice. The aging model included three age groups (6 mice per group): 6 months (youth), 12 months (middle age), and 18 months (old age). The DMM model had two groups (6 mice per group): Sham and DMM. For the DMM model, 12‐week‐old mice received 129 intraperitoneal injections of 3% pentobarbital sodium (20 mg/kg). A 5‐mm incision was made in the right knee to expose and destabilize the medial meniscus by removing the ligament attached to its anterior horn.

To study CARM1's role, CARM1 inhibitor (6 μL of 5 ng/mL; Med Chem Express) and recombinant lentivirus (6 μL of 10^8^ PFU/mL; Hanbio) for CARM1 over‐expression were injected into the intra‐articular cavity of 12‐week‐old male C57BL/6 mice after DMM. Mice were divided into groups: Sham, DMM, and DMM + CARM1 inhibitor (*n* = 6/group) for the inhibitor study, and Sham, DMM, DMM + LV‐NC, DMM + LV‐CARM1, and DMM + LV‐CARM1 + CARM1 inhibitor (*n* = 6/group) for the over‐expression study. Using the random number table method to assign mice into groups, the authors conducting the experiment are aware of the group information after the assignment. All mice were adaptively housed for two weeks in a specific pathogen‐free (SPF) environment before the intervention. If any animals die unexpectedly during the experiment, they should be excluded, and the corresponding group should be promptly supplemented with new animals according to the original plan.

### 2.3 Plain Radiography

After the intervention period, C57BL/6 mice were sacrificed with excessive pentobarbital sodium to separate knee joint samples. The knee joint of C57BL/6 mice was fixed on the tray, and plain knee radiographs were taken with an X‐ray machine (Faxitron). Finally, image evaluation was performed according to osteophyte formation and joint space stenosis.

### 2.4 Culture of Human Cartilage Explants

We collected cartilage explants from the tibial plateau of patients who underwent traumatic amputation. The explants were fragmented into approximately 5‐mm^3^ pieces and cultured in DMEM/F12 medium with 10% fetal bovine serum, supplemented with IL‐1β (20 ng/mL) or a CARM1 inhibitor (5 ng/mL). The medium was refreshed every two days. After 2 weeks, the cartilage explants were collected for histological analysis.

### 2.5 Primary Articular Chondrocyte Preparation and Culture

Under sterile conditions, we extracted knee articular cartilage from 6‐day‐old C57BL/6 mice. The cartilage was enzymatically digested in DMEM/F12 (HyClone, Logan, UT, USA) with 2 mg/mL type II collagenase (Invitrogen, Carlsbad, CA, USA) for 2 h. The cell suspension was filtered and cultured in DMEM/F12 with 10% fetal bovine serum (Life Technologies, Carlsbad, CA, USA). After 72 h, the medium was changed, and chondrocytes were subcultured at 90% density. Isolated chondrocytes were identified using Safranin O, Alcian Blue, and COL2A1 immunohistochemistry staining.

In some experiments, primary chondrocytes and ATDC5 cells were treated with IL‐1β, and ATDC5 cells were also treated with a CARM1 inhibitor or recombinant lentivirus for CARM1 over‐expression.

### 2.6 Western Blotting Analysis

Cellular proteins were extracted using RIPA lysis buffer (AR0105, Boster), separated by SDS‐PAGE (EpiZyme Biotechnology, China), and transferred onto PVDF membranes (PVDF, HVLP09050, Millipore). Membranes were incubated with primary antibodies overnight at 4°C, followed by secondary antibodies for 1 h at room temperature. Visualization was performed using the BIO‐RAD CHEMIDoc XRS+ system. Antibodies used included COL2A1(Abcam; 1:1000), ACAN(Abcam; 1:1000), MMP13(Abcam; 1:1000), CARM1 (all 1:1000, Abcam), Caspase3 (Santa Cruz Biotechnology; 1:500), ERK1/2(Cell Signaling Technology; 1:1000), p‐ERK1/2(Cell Signaling Technology; 1:1000), and GAPDH (Cell Signaling Technology; 1:1000).

### 2.7 RNA Extraction and RT‐qPCR Analysis

Total RNA was extracted using Trizol (Beyotime, Shanghai), and the extracted RNA was then reverse‐transcribed into cDNA to detect the mRNA expression. Samples were evaluated by RT‐qPCR on a real‐time fluorescence quantitative PCR apparatus (QuantStudio 6 Flex). Using GAPDH as the reference value, the expression of ACAN, CARM1, MMP13, Caspase3, and COL2A1 was assessed. All RT‐qPCRs were set up in three duplicate wells. Table S1 lists the primers, and 2^−ΔΔCt^ is the fold difference between the target gene's expression in the experimental group and the control group.

### 2.8 Histology and Immunohistochemistry

Human cartilage and mouse whole knee joints were fixed with 4% paraformaldehyde (PFA) for 2 days and decalcified with EDTA for 3 weeks before paraffin embedding. Sections (5‐μm thick) were deparaffinized, hydrated, and stained with Safranin O‐Fast Green and Alcian Blue to evaluate cartilage destruction. The cartilage integrity was scored using the OARSI scale (0–6), with 0 indicating no damage and 6 representing complete cartilage loss. Additionally, the Mankin score (0–14) was employed to assess more detailed cartilage damage, including surface irregularities, matrix loss, chondrocyte alterations, and subchondral bone changes. For both scoring systems, two independent observers conducted the assessment, and the final score was averaged to ensure consistency. TUNEL staining detected apoptosis in mouse knee chondrocytes and synovial cells. Immunohistochemistry (IHC) staining on human and mouse cartilage sections identified CARM1(Abcam; 1:100), ACAN(Abcam; 1:100), and MMP13 (Abcam; 1:100).

### 2.9 Immunofluorescence Analysis

Mouse primary chondrocytes were rinsed with PBS and fixed with 4% paraformaldehyde for 15 min at room temperature. Cells were permeabilized with 1% Triton X‐100 for 20 min and blocked with 5% BSA (Beyotime, Shanghai) for 1 h. They were then incubated with primary antibodies against ACAN(Abcam; 1:100), CARM1(Abcam; 1:100), and MMP13(Abcam; 1:100) overnight at 4°C. The next day, fluorescent secondary antibodies were added and incubated for 1 h in a dimly lit environment, followed by DAPI (AR1176, Boster) staining for 30 min. Human articular cartilage and mouse knee joint sections were dewaxed, hydrated, and subjected to immunofluorescence staining using a multiplex kit (Absin, Shanghai). Finally, slices were sealed and imaged under a microscope.

### 2.10 Cell Counting Kit‐8 (CCK8) Assay

CCK‐8 technique was used to detect cell proliferation. ATDC5 transfected with LV‐NC or LV‐CARM1 and interfered with IL‐1β or CARM1 inhibitor was inoculated in 96‐well plates at a density of 4000 cells per well. The proliferation of ATDC5 was then evaluated at 0, 1, 2, and 3 days according to the instructions of the CCK‐8 kit (AR1160, Boster). Moreover, 10 μL of CCK‐8 solution was added to each well and incubated for 1 h at 37°C in the dark. On days 0, 1, 2, and 3, the optical density (OD) values were then determined at 450 nm and recorded.

### 2.11 TUNEL Staining

The One Step TUNEL Apoptosis Assay Kit (Beyotime, Shanghai) was used to identify apoptosis in ATDC5. ATDC5 transfected with LV‐NC or LV‐CARM1 was inoculated into 6‐well plates with cell crawlers. After adherence, the prepared cell crawls were removed, washed with PBS, fixed with 4% PFA for 15 min, and permeabilized with 1% X‐Triton for 20 min. Following the TUNEL Apoptosis Detection Kit's instructions, cells were stained red to demonstrate the degree of apoptosis. Besides, different regions on the slides were randomly chosen and imaged under a fluorescence microscope to count the number of TUNEL‐positive cells.

### 2.12 Flow Cytometry Analysis

ATDC5 cell apoptosis levels were detected using the Annexin V‐FITC/PI Double Staining Kit (E‐CK‐A211, Elabscience). ATDC5 transfected with LV‐NC or LV‐CARM1 and interfered with IL‐1β or CARM1 inhibitors were collected with EDTA‐free trypsin, rinsed with PBS, and handled according to kit instructions. Simply, suspend the cells in 100 μL of binding buffer, place them in a centrifuge tube, add 2.5 μL of dye, and incubate for 30 min in the dark. Finally, add 400 μL of binding buffer to the tube for flow cytometry detection.

### 2.13 5‐Ethynyl‐2′‐Deoxyuridine (EdU) Assay

Following the ATDC5 intervention, the assessment of cell proliferation was performed using the BeyoClick EdU‐488 Cell Proliferation Kit (Beyotime, Shanghai). The chondrocytes were incubated with a diluted EdU solution in the culture media at a ratio of 1:1000 for a duration of 2 h. Subsequently, the cells were fixed with a 4% paraformaldehyde (PFA) solution for 15 min and permeabilized using 1% X‐Triton for 15 min. To perform EdU staining, the staining solution was prepared according to the instructions provided with the kit and incubated with the cells for 30 min at room temperature in a dark environment. Finally, the nuclei were stained with DAPI for 10 min and observed using a fluorescence microscope.

### 2.14 Statistical Analysis

Referencing the common practices in most literature and ensuring statistical significance, each group consisted of 6 mice. A minimum of three repetitions was carried out for all experiments, and the data were presented as the mean ± standard deviation (SD). Data analysis was performed utilizing GraphPad Prism 8 software (San Diego, CA, USA) or SPSS 18.0 statistical software (IBM Corp). Unpaired two‐tailed Student's *t*‐tests were employed for comparisons between two groups, while one‐way ANOVA tests were utilized for comparisons involving multiple groups. Statistical significance was established at a threshold of *p* < 0.05.

## 3 Results

### 3.1 Up‐Regulation of CARM1 in Articular Cartilage and Chondrocytes With OA

**FIGURE 1:**
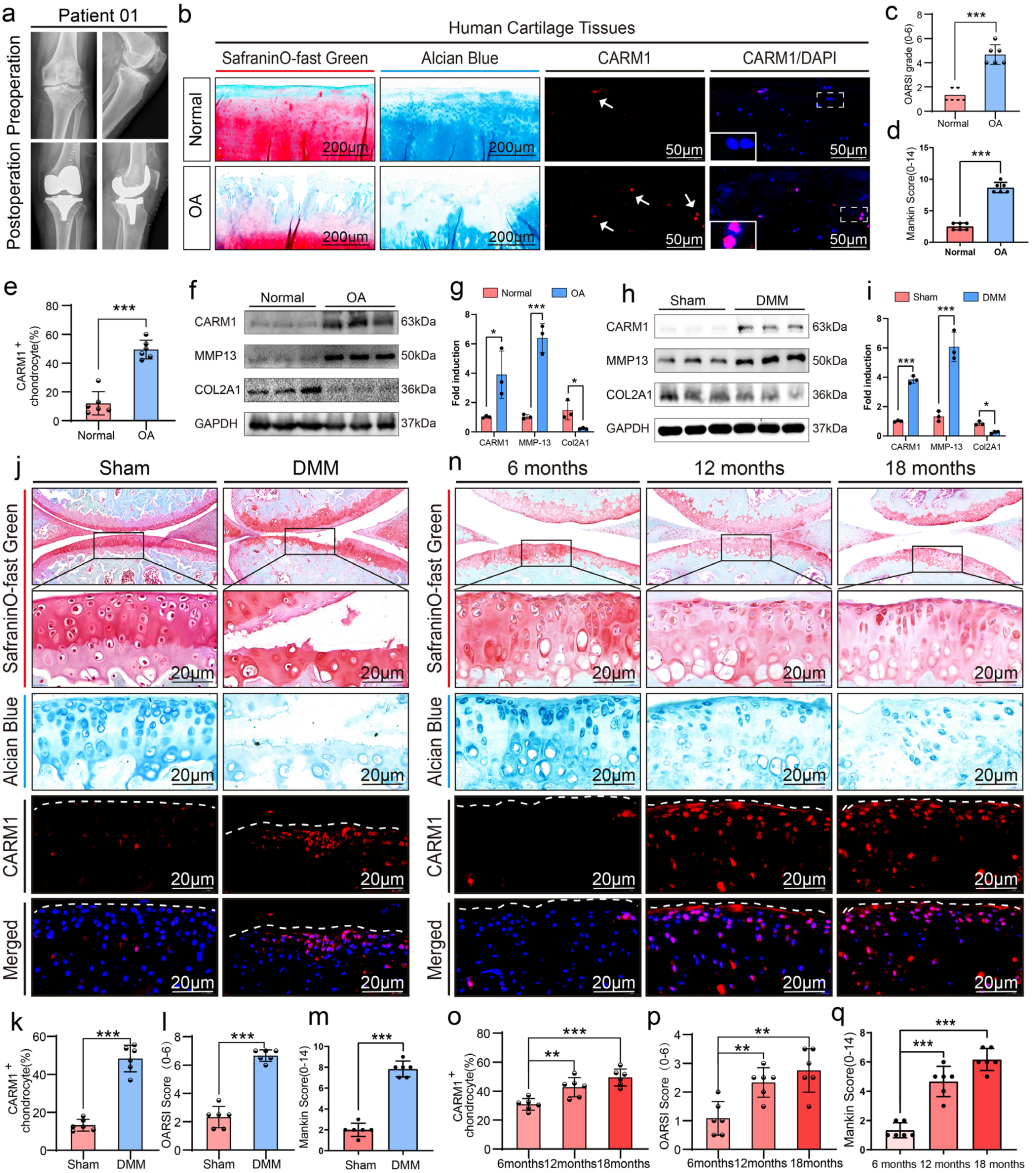
CARM1 is up‐regulated in the articular cartilage of osteoarthritis patients, osteoarthritis mice and aged mice. (a) Clinical preoperative and postoperative X‐ray plain film scanning of patients. (b–e) Safranin O‐Fast Green, Alcian Blue, CARM1 Immunofluorescent staining, Statistical analysis of the percentage of CARM1^+^ chondrocytes, the OARSI grade, and Mankin score of normal and OA cartilage tissues (*n* = 6 per group). (f, g) Western blot analysis and quantification of CARM1, MMP13, and COL2A1 in normal and OA cartilage tissues, with GAPDH as the endogenous control. (h, i) Western blot analysis and quantification of CARM1, MMP13, and COL2A1 in Sham and DMM groups, with GAPDH as the endogenous control. (j–m) Safranin O‐Fast Green, Alcian Blue, and CARM1 Immunofluorescent staining, and Statistical analysis of the percentage of CARM1^+^ chondrocytes and the OARSI and Mankin score of cartilage from Sham and DMM groups (*n* = 6 per group). (n–q) Safranin O‐Fast Green staining, Alcian Blue staining, and CARM1 immunofluorescent staining were performed on cartilage samples from the 6‐, 12‐, and 18‐month groups (*n* = 6 per group), followed by statistical analysis of the percentage of CARM1^+^ chondrocytes, as well as OARSI and Mankin scores. Data are presented as the mean ± SD; *p < 0.05, **p < 0.01, ***p < 0.001.

By analyzing RNA sequencing data(GSE103416) related to degenerated cartilage from the GEO database, we found that CARM1 is highly expressed in osteoarthritic cartilage tissue(Figure S1). To assess CARM1's role in OA, we collected clinical images (Figure 1a) and used Safranin O‐Fast Green, Alcian Blue, Immunofluorescence staining, OARSI scoring, and Mankin scores. Normal cartilage was smooth, while OA samples had cracks and fissures, with significantly upregulated CARM1 (Figure 1b–e). Western blotting showed increased levels of CARM1, MMP13, and COL2A1 in OA cartilage (Figure 1f,g). In aging and DMM‐induced mouse OA models, the successful establishment of the models was confirmed by staining, OARSI scoring and Mankin scores. Both Western blotting (Figure 1h,i) and immunofluorescence analysis (Figure 1j–m) demonstrated that the expression of CARM1 was higher in the DMM group compared to the normal control group. OARSI scores, Mankin scores, and CARM1 levels were also higher in 12‐ and 18‐month‐old mice compared to 6‐month‐old mice (Figure 1n–q).

In primary chondrocytes from OA mice, Safranin O, Alcian Blue, and COL2A1 IHC staining confirmed type II collagen‐positive cells (Figure S2a–c). IL‐1β‐treated OA model mice showed increased protein and mRNA levels of CARM1, MMP13, and COL2A1, with CARM1 expression rising with higher IL‐1β concentrations (Figure S3a–c). Immunofluorescence analysis also revealed elevated CARM1 levels in IL‐1β‐treated chondrocytes (Figure S3d,e). Our findings demonstrated that OA leads to CARM1 accumulation in chondrocytes and articular cartilage.

### 3.2 Inhibition of CARM1 Rescued OA‐Related Degeneration in IL‐1β‐Induced ATDC5

**FIGURE 2:**
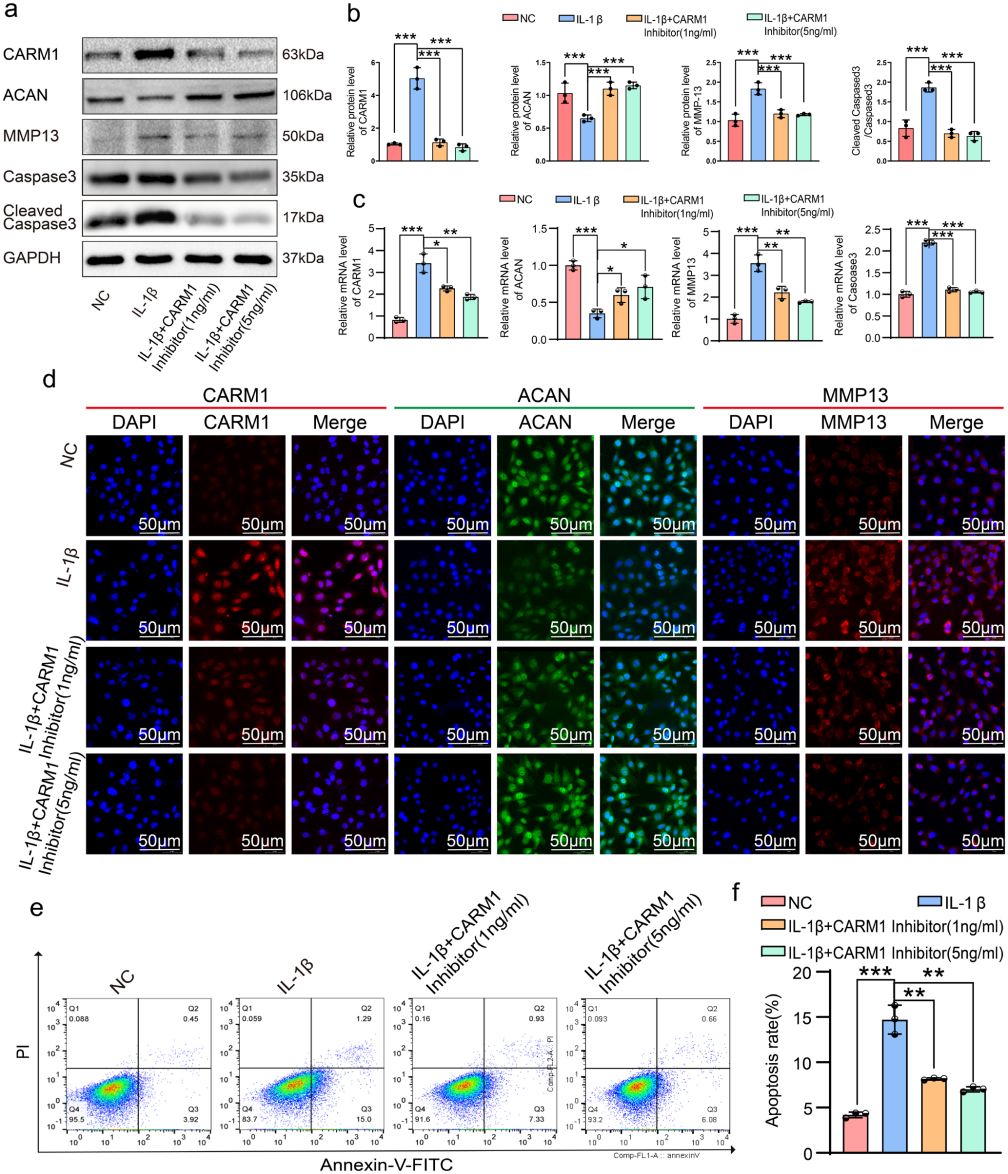
Inhibition of CARM1 inhibited the decrease of anabolism, the increase of catabolism and the apoptosis of ATDC5 induced by IL‐1β. (a–c) The protein and mRNA expression levels of CARM1, MMP13, ACAN, Cleaved Caspase3 and Caspase3 were measured by Western blot, densitometric quantification of Western blot, and RT‐qPCR assay (without Cleaved Caspase3) in ATDC5 cells treated with IL‐1β (20 ng/mL) or CARM1 inhibitor (1 or 5 ng/mL) for 24 h. (d) CARM1, ACAN and MMP13 immunofluorescent staining of ATDC5 treated with IL‐1β (20 ng/mL) or CARM1 inhibitor (1 or 5 ng/mL) for 24 h. (e, f) Apoptotic ATDC5 stained by annexin V and PI and analyzed by flow cytometry after treatment with IL‐1β (20 ng/mL) or CARM1 inhibitor (1 or 5 ng/mL) for 24 h, ATDC5 without treatment were used as the negative control. Data are presented as the mean ± SD; *p < 0.05, **p < 0.01, ***p < 0.001.

To investigate CARM1's role in OA treatment, ATDC5 cells with OA (stimulated with 20 ng/mL IL‐1β) were treated with a CARM1 inhibitor (Figure S4a). CCK8 data showed low concentrations of the inhibitor (0.1, 1.0, 5.0 ng/mL) had little effect on ATDC5, while higher concentrations (10, 20, 40 ng/mL) significantly inhibited cell proliferation at 24 and 48 h (Figure S4b–d). Western blot (WB), RT‐qPCR, and immunofluorescence confirmed CARM1 inhibition reduced its expression and increased anabolic marker ACAN, while catabolic marker MMP13 and apoptosis marker Cleaved Caspase3/Caspase3 decreased (Figure 2a–d). Flow cytometry and EdU staining indicated reduced apoptosis (Figure 2e,f) and increased proliferation (Figure S5a,b) following CARM1 inhibition. These findings suggest that CARM1 inhibition mitigates OA‐related degeneration in IL‐1β‐induced ATDC5 cells.

### 3.3 Inhibition of CARM1 Enhanced Anabolism and Inhibited Catabolism in IL‐1β‐Treated Cartilage Explants

To determine whether inhibition of CARM1 prevented OA‐related degradation of IL‐1β‐treated cartilage explants, knee cartilage samples from individuals undergoing traumatic amputations were cultured for 2 weeks (Figure S6a). After 2 weeks of culture, cartilage explants were stained with Safranin O‐Fast Green and Alcian Blue to evaluate the proteoglycan content. The results showed that IL‐1β treated histonean content was significantly reduced compared to the control group, while most of the recovery of CARM1 histonean content was inhibited (Figure S6b). Two weeks after culture, immunohistochemical staining showed a decrease in CARM1, which significantly increased ACAN concentration and reduced MMP13 levels (Figure S6c–f).

### 3.4 Inhibition of CARM1 Attenuated OA‐Related Degeneration in DMM‐Induced Mice

**FIGURE 3:**
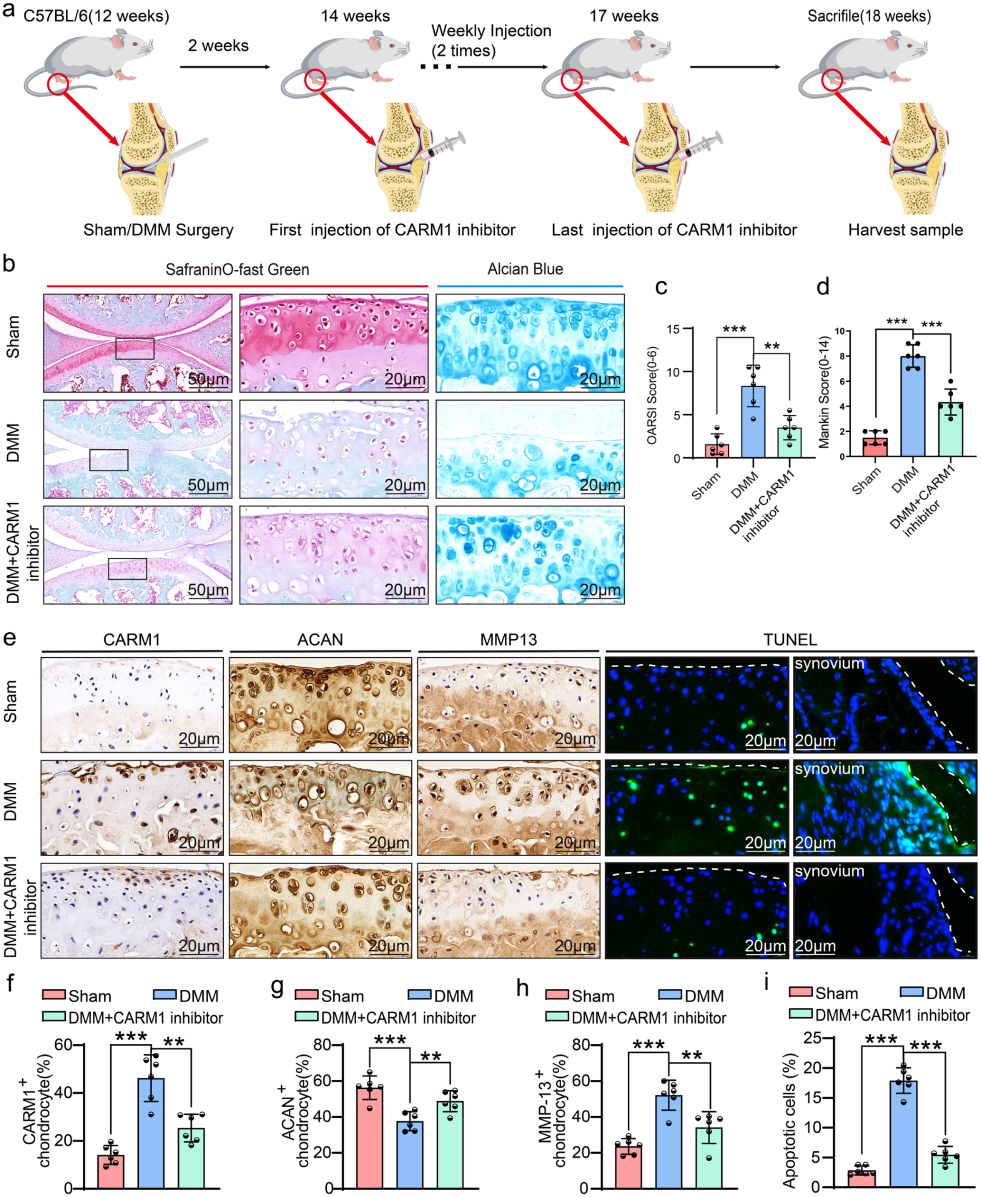
Inhibition of CARM1 attenuated DMM‐induced osteoarthritis development. (a) Schematic diagram of the process of building OA model and inhibiting CARM1 in mice. (b–d) Representative images of Safranin O‐Fast Green and Alcian Blue staining, with the corresponding OARSI and Mankin scores, were collected from Sham, DMM, and DMM + CARM1 inhibitor groups (*n* = 6 per group). (e) IHC staining of CARM1, ACAN and MMP13 and TUNEL staining from Sham, DMM, and DMM + CARM1 inhibitor groups (*n* = 6 per group). (f–i) Statistical analysis of the percentage of CARM1^+^, ACAN^+^, MMP13^+^, and apoptotic chondrocytes in articular cartilage of samples shown in D. Data are presented as the mean ± SD; *p < 0.05, **p < 0.01, ***p < 0.001.

We investigated whether intra‐articular injection of a CARM1 inhibitor could alleviate OA progression post‐DMM in mice. Based on in vitro experiments, C57BL/6 mice received twice‐weekly injections of CARM1 inhibitor (5.0 ng/mL) for 4 weeks (Figure 3a). Safranin O‐Fast Green and Alcian Blue staining revealed notable proteoglycan preservation in the CARM1 inhibitor‐treated group compared to vehicle‐treated DMM controls (Figure 3b). CARM1 inhibitor significantly suppressed articular cartilage erosion, as confirmed by lower OARSI and Mankin scores (Figure 3c,d). IHC results showed increased ACAN levels and decreased CARM1 and MMP13 levels in the inhibitor‐treated group. TUNEL staining indicated significantly reduced apoptosis in chondrocytes and synovial cells (Figure 3e–i). Overall, CARM1 inhibitor‐treated knees exhibited fewer osteoarthritic changes than vehicle‐treated knees.

### 3.5 CARM1 Over‐Expression Aggravated OA‐Related Degeneration in ATDC5

**FIGURE 4:**
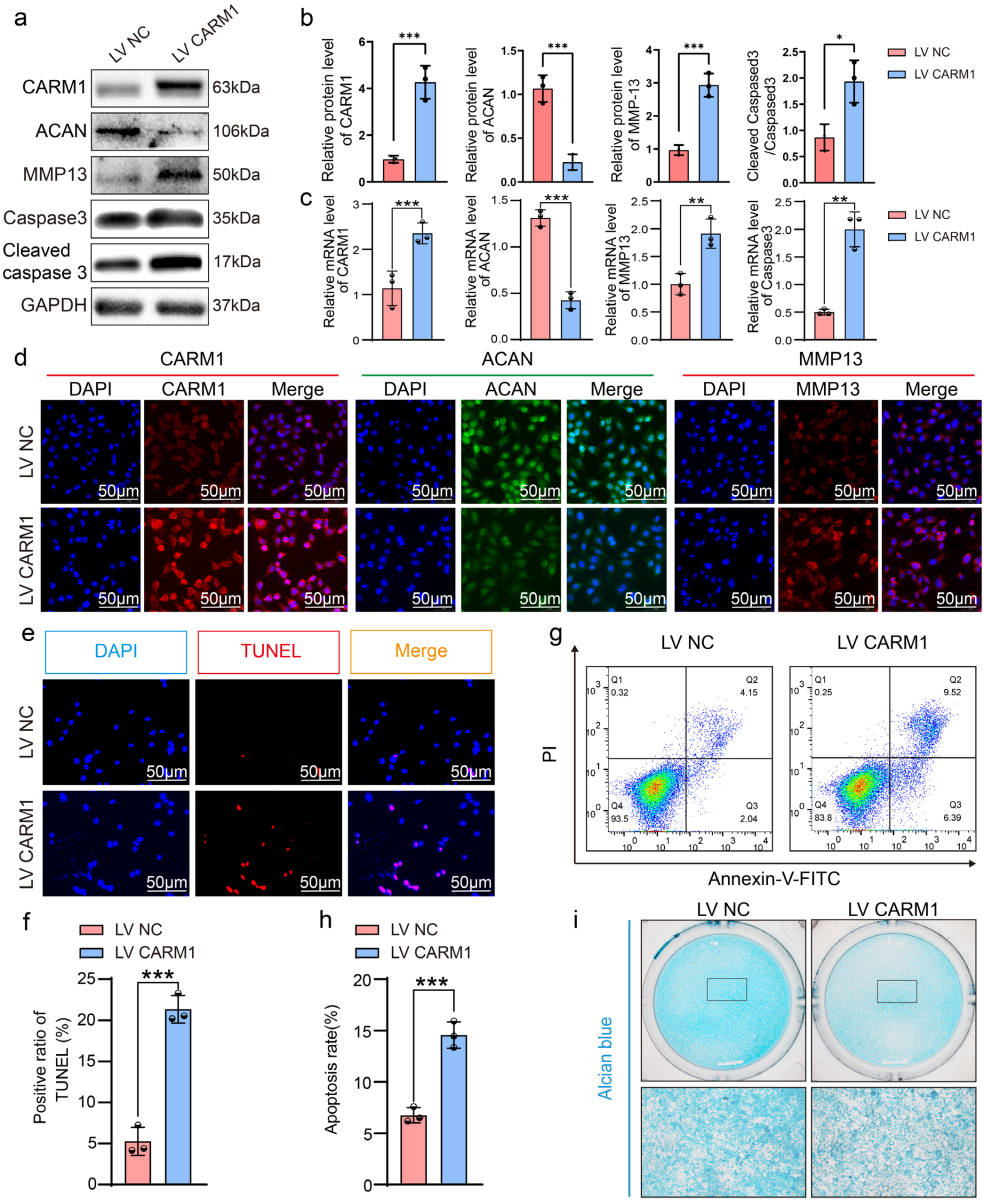
Over‐expression of CARM1 aggravated the decrease of anabolism, the increase of catabolism and the apoptosis of ATDC5. (a, b) The protein levels of CARM1, MMP13, ACAN, Cleaved Caspase3 and Caspase3 were detected and quantified by WB assay in ATDC5 transfected with CARM1 over‐expression lentivirus. (c) The mRNA levels of apoptosis marker Caspase3 and ECM‐related biomarkers ACAN and MMP13 in ATDC5 transfected with CARM1 over‐expression lentivirus. (d) CARM1, ACAN, and MMP13 immunofluorescent staining of ATDC5 transfected with CARM1 over‐expression lentivirus. (e) Representative images show TUNEL staining assay in ATDC5 transfected with CARM1 over‐expression lentivirus. (f) Quantitative analysis shows the total numbers of TUNEL positive in ATDC5 transfected with CARM1 over‐expression lentivirus. (g) Flow cytometry analysis and (h) quantification in ATDC5 transfected with CARM1 over‐expression lentivirus. (i) Representative images show Alcian Blue staining assay in ATDC5 transfected with CARM1 over‐expression lentivirus. Data are presented as the mean ± SD; *p < 0.05, **p < 0.01, ***p < 0.001.

ATDC5 cells were transfected with lentiviral overexpression of CARM1. Overexpression efficiency and related markers were confirmed by WB, RT‐qPCR, and immunofluorescence. CARM1 expression significantly increased, while the anabolic marker ACAN decreased. Conversely, the catabolic marker MMP13 and the apoptotic marker Cleaved Caspase3/Caspase3 were upregulated (Figure 4a–d). Flow cytometry and TUNEL staining showed increased apoptosis with CARM1 overexpression (Figure 4e–h). CCK8 and EdU staining indicated that CARM1 overexpression significantly inhibited ATDC5 proliferation (Figure S7a–c). Alcian Blue staining revealed decreased proteoglycan content in ATDC5 cells (Figure 4i).

### 3.6 CARM1 Over‐Expression Exacerbated OA‐Related Degeneration in DMM‐Induced Mice

**FIGURE 5:**
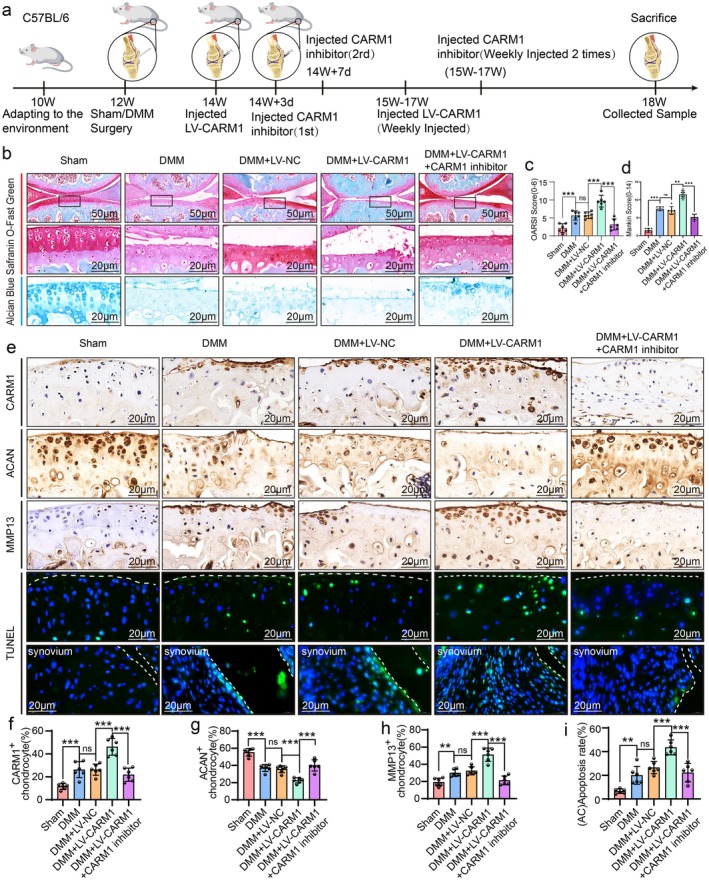
CARM1 over‐expression exacerbated OA‐related degeneration in DMM‐induced mice. (a) Schematic diagram of the process of building OA model and over‐expressing CARM1 in mice. (b) Representative images of Safranin O‐Fast Green and Alcian Blue staining and (c, d) the corresponding OARSI and Mankin scores from Sham, DMM, DMM + LV‐NC, DMM + LV‐CARM1, and DMM + LV‐CARM1 + CARM1 inhibitor groups (*n* = 6 per group). (e) IHC staining of CARM1, ACAN and MMP13 and TUNEL staining from Sham, DMM, DMM + LV‐NC, DMM + LV‐CARM1, and DMM + LV‐CARM1 + CARM1 inhibitor groups (*n* = 6 per group). (f–i) Statistical analysis of the percentage of CARM1^+^, ACAN^+^, MMP13^+^, and apoptotic chondrocytes in articular cartilage of samples shown in D. Data have presented as the mean ± SD; ns: not significant, *p < 0.05, **p < 0.01, ***p < 0.001.

To further determine CARM1's role in OA treatment, C57BL/6 mice were injected with CARM1 overexpression lentivirus (Figure 5a). X‐ray images showed that the DMM and LV‐NC groups exhibited osteoarthritis symptoms and joint space narrowing, which was severe in the LV‐CARM1 group. The CARM1 inhibitor group showed recovery from joint space narrowing compared to the LV‐CARM1 group (Figure S8a,b). Staining results indicated that CARM1 overexpression significantly increased knee cartilage degeneration, decreased cartilage thickness, and increased both OARSI and Mankin scores (Figure 5b–d). IHC results revealed that CARM1 overexpression increased CARM1 and MMP13 levels while reducing ACAN levels, compared to the DMM and LV‐NC groups. TUNEL staining showed increased apoptosis in chondrocytes and synovial cells with CARM1 overexpression, which was rescued by the CARM1 inhibitor (Figure 5e–i).

### 3.7 CARM1‐Mediated ERK1/2 Phosphorylation and Nuclear Translocation in Osteoarthritic Chondrocytes

To investigate the molecular mechanisms underlying CARM1‐mediated cartilage degeneration in OA, we constructed a Flag‐tagged CARM1 overexpression lentivirus in this study. Through immunoprecipitation, we analyzed the co‐precipitated proteins by mass spectrometry (Table S2). We specifically identified proteins that were detected only in the overexpression group and not in the control group, including MAPK1 and MAPK3 (i.e., ERK1/2). Additionally, through protein–protein interaction (PPI) analysis, we found that CARM1 can interact with ERK1/2 (Figure S9).

**FIGURE 6:**
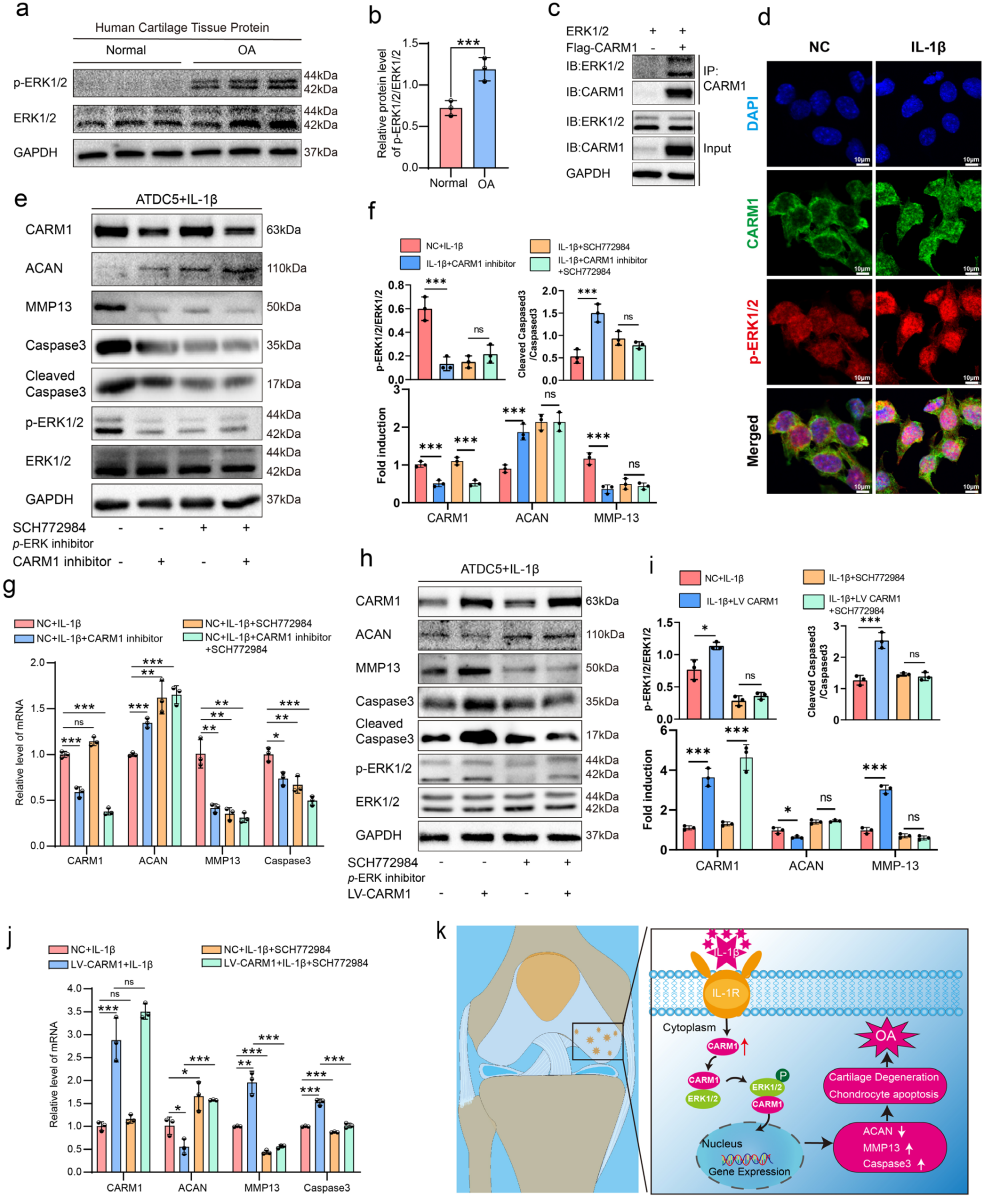
CARM1 accelerates OA‐related degeneration by interacting and phosphorylating ERK1/2. (a, b) Western blotting and quantification of ERK1/2 and p‐ERK1/2 in normal and OA cartilage tissues, with GAPDH as the endogenous control. (c) CARM1 and ERK1/2 proteins immunoprecipitated from ATDC5 with anti‐CARM1 and anti‐ERK1/2 antibodies, respectively, were analyzed with WB. (d) After IL‐1β (20 ng/mL) treatment of ATDC5 cells, immunofluorescence co‐localization revealed the expression and localization of CARM1 (green) and p‐ERK1/2 (red). (e–g) Western blotting, densitometric quantification of Western blot and RT‐qPCR (without Cleaved Caspase3) analysis shows the levels of ERK1/2, p‐ERK1/2, CARM1, ACAN, MMP13, Cleaved Caspase3 and Caspase3 proteins in IL‐1β‐treated ATDC5 treated with 5 ng/mL CARM1 inhibitor, 0.5 μM SCH772984 (ERK1/2 inhibitor) or co‐treatment with CARM1 inhibitor and 0.5 μM SCH772984. (h–j) Western blotting and RT‐qPCR (without Cleaved Caspase3) analysis show the levels of ERK1/2, p‐ERK1/2, CARM1, ACAN, MMP13, Cleaved Caspase3, and Caspase3 in IL‐1β‐treated ATDC5 treated with CARM1 over‐expression lentivirus, 0.5 μM SCH772984 (ERK1/2 inhibitor) or co‐treatment with CARM1 over‐expression lentivirus and 0.5uM SCH772984. (k) A graphical representation of the hypothesized mechanism by which CARM1 alleviates OA. Data are presented as the mean ± SD; ns: not significant, *p < 0.05, **p < 0.01, ***p < 0.001.

Meanwhile, we examined the expression of ERK1/2 and phosphorylated ERK1/2 (p‐ERK1/2) in human OA cartilage tissue and relatively normal cartilage tissue, revealing a significantly increased phosphorylation level of ERK1/2 in OA cartilage (Figure 6a,b). Furthermore, in primary mouse chondrocyte cultures, we observed that chondrocytes at passage 4, indicative of replication‐induced senescence, exhibited increased senescence markers, such as p16 and p21, compared to the initial primary chondrocytes, along with significantly elevated expression levels of ERK1/2 and p‐ERK1/2 (Figure S10a,b). Stimulation of chondrocytes with other inflammatory cytokines (TNF‐α, IL‐6) also led to a marked increase in the expression of ERK1/2 and p‐ERK1/2, indicating activation of the ERK1/2 pathway in degenerative chondrocytes (Figure S10c–f). Finally, we performed Co‐IP verification and confirmed that CARM1 indeed interacts with ERK1/2 (Figure 6c). To investigate the downstream effects of CARM1‐ERK1/2 interaction, we performed immunofluorescence staining to assess the co‐localization of CARM1 and phosphorylated ERK1/2 (p‐ERK1/2). Compared with the control group, IL‐1β stimulation led to increased expression of both CARM1 and p‐ERK1/2, along with enhanced nuclear translocation. These findings suggest that CARM1 promotes ERK1/2 phosphorylation and facilitates their co‐translocation into the nucleus (Figure 6d).

### 3.8 CARM1 Regulates Chondrocyte Apoptosis and Anabolic/Catabolic Balance Through ERK1/2 Phosphorylation

ERK1/2 is a key marker of the MAPK pathway, and in addition to ERK1/2, JNK, and P38 also play important roles in the MAPK signaling cascade. To assess the effect of CARM1 modulation on JNK and P38 in the MAPK pathway, we performed Western blot analysis. The results showed that, apart from p‐ERK1/2, neither inhibition nor overexpression of CARM1 had any significant effect on JNK, P38, or their phosphorylated forms (p‐JNK and p‐P38). This suggests that CARM1 specifically regulates the activation of ERK1/2 (Figure S11).

To confirm whether CARM1 exerts its protective effects on chondrocyte degeneration through the regulation of ERK1/2 phosphorylation, we used the specific inhibitor of p‐ERK1/2, SCH772984, for intervention. After inducing degeneration in ATDC5 cells with IL‐1β, subsequent inhibition of CARM1 was followed by Western blot and RT‐qPCR assays, which revealed an increase in anabolic markers and a decrease in catabolism and apoptosis. However, when ERK1/2 phosphorylation was inhibited using the p‐ERK1/2 inhibitor, the increase in anabolic markers and the reduction in catabolism and apoptosis observed after CARM1 inhibition were diminished. This suggests that blocking ERK1/2 phosphorylation can attenuate the protective effects of CARM1 inhibition (Figure 6e–g). Similarly, CARM1 overexpression was found to enhance catabolism and apoptosis while reducing anabolic activity. Yet, after inhibition of ERK1/2 phosphorylation, the changes in catabolic and anabolic activities induced by CARM1 overexpression were also weakened (Figure 6h–j). These findings suggest that CARM1 exerts its effects through the regulation of ERK1/2, highlighting its novel role in regulating chondrocyte apoptosis and OA progression via this pathway. Ultimately, by interacting with ERK1/2 and facilitating its phosphorylation, targeting CARM1 may offer a potential therapeutic strategy for mitigating OA (Figure 6k).

## 4 Discussion

OA is a chronic inflammatory condition affecting the entire joint, not just cartilage degeneration (Latourte et al. 2017). Its complex pathogenesis involves multiple pathways, including ERK1/2, apoptosis, proliferation, autophagy, anabolism, and catabolism (Kim, Jeon, et al. 2014; Ji et al. 2019; Zhang et al. 2017; Willcockson et al. 2021). While CARM1 is involved in various biological processes across different cell types and diseases, its role in OA remains largely unknown. Tatsuo et al. reported that CARM1 regulates chondrocyte proliferation via Sox9 arginine methylation (Ito et al. 2009). Layla et al. demonstrated CARM1's regulatory role in 25‐Hydroxyvitamin D3 24‐Hydroxylase, an enzyme in bone catabolism (Panach et al. 2020). This study aimed to examine CARM1's impact on OA progression using a DMM mouse model.

Cartilage‐resident progenitor cells aid cartilage repair by differentiating into chondrocytes, which release ECM components and regulate cartilage metabolism (Camarero‐Espinosa et al. 2016). Mature chondrocytes secrete aggrecan (ACAN) and type II collagen (COL2A1), crucial for cartilage stability and function. Dysfunction in chondrocytes is linked to OA‐related cartilage damage (Roseti et al. 2019), primarily due to imbalances in tissue remodeling from altered chondrocyte activity (Cohen‐Solal et al. 2012). During OA progression, chondrocytes produce excessive ECM‐degrading enzymes like ADAMTS and MMPs, leading to ECM destruction (Chen, Hu, et al. 2017). ADAMTS degrades ACAN, while MMP13 targets COL2A1 (Goldring 2012), exposing subchondral bone and causing a rough cartilage surface (Goldring and Goldring 2010). Inhibiting ADAMTS and MMP13 slows OA progression by preserving ACAN and COL2A1, maintaining ECM homeostasis and cartilage integrity (Little et al. 2009). This study observed significant up‐regulation of CARM1 in degenerative cartilage, aging, DMM‐induced mouse OA models, human OA patients, and IL‐1β‐treated chondrocytes. In vitro and in vivo experiments with CARM1 inhibitors and overexpression lentiviruses showed that CARM1 inhibition suppressed OA‐related cartilage degeneration by increasing ACAN expression (anabolism), reducing apoptosis (lower Caspase3), and decreasing MMP13 expression (catabolism). Conversely, CARM1 overexpression promoted OA‐related degeneration. These findings suggest CARM1 regulates OA progression by modulating chondrocyte ECM synthesis and degradation.

Apoptosis of chondrocytes is closely associated with the pathogenesis of OA (Yan et al. 2016). The pathophysiology of OA involves a combination of factors, including metabolic changes, mechanical stress, and inflammation, all of which contribute to chondrocyte apoptosis (Hwang and Kim 2015). Prior studies have indicated that chondrocytes undergo apoptosis during OA development as a result of elevated levels of reactive oxygen species (ROS) and oxidative stress (Hwang and Kim 2015). Excessive accumulation of ROS triggers the release of activated mitochondrial apoptotic proteins, leading to mitochondrial outer membrane permeabilization (MOMP), which ultimately activates cellular apoptosis (Vakifahmetoglu et al. 2006). Dong‐il et al. reported that high glucose‐induced CARM1 expression increases apoptosis in retinal pigment epithelial (RPE) cells through asymmetric dimethylation of H3R17, and reducing CARM1 expression can be used to prevent RPE apoptosis in the progression of diabetic retinopathy (Kim, Park, et al. 2014).

A study by Bing et al. demonstrated that elevated expression of CARM1 suppresses the migration and proliferation of lung cancer (LC) cells and induces apoptosis in LC cells (Hu et al. 2020). However, the role of CARM1 in chondrocyte apoptosis remains unknown. In our current study, we observed a significant decrease in the number of TUNEL‐positive staining and a reduction in the levels of Caspase3, a protein associated with cellular apoptosis, in IL‐1β‐treated ATDC5 cells with inhibited expression of CARM1. These findings suggest a potential involvement of CARM1 in modulating chondrocyte apoptosis. On the contrary, over‐expressed ATDC5 of CARM1 showed an increase in TUNEL‐positive staining and the level of apoptosis‐related protein Cleaved Caspase3. Furthermore, we observed a reduction in TUNEL‐positive staining in knee articular cartilage and synovium sections from mice in the DMM + CARM1 inhibitor group compared to those from mice in the DMM group. Conversely, mice in the LV‐CARM1 group exhibited an increase in TUNEL‐positive staining in knee cartilage and synovial sections compared to mice in the LV‐NC group. These findings provide evidence supporting the role of CARM1 in mediating chondrocyte apoptosis during the progression of OA in knee articular cartilage tissues.

In order to gain insight into the precise molecular mechanism underlying the regulation of cartilage degeneration by CARM1, we conducted a Co‐Immunoprecipitation (Co‐IP) assay and identified ERK1/2 as one of the binding proteins of CARM1. ERK1/2 belongs to the same pathway as P38 and JNK, which could be activated by CARM1 (Suresh et al. 2021; Shirley et al. 2009). Next, we confirmed that inhibition of CARM1 inactivates the ERK1/2 pathway, whereas its overexpression activates the said pathway. The ERK1/2 signaling pathway regulates chondrocyte apoptosis, matrix degradation, and activation of mature genes (Miedlich et al. 2010; Ding et al. 2010). Susanne et al. provided evidence that apoptosis of chondrocytes induced by phosphate is linked to a decline in mitochondrial membrane potential and is reliant on the phosphorylation of ERK1/2 (Miedlich et al. 2010). Indira et al. demonstrated that activation of the ERK1/2 signaling pathway enhances the synthesis of ADAMTS and MMPs in articular chondrocytes, resulting in cartilage degeneration (Prasadam et al. 2012). Based on these findings, we hypothesized that CARM1 regulates cartilage degeneration through modulation of the ERK1/2 signaling pathway. We observed that inhibiting the expression of p‐ERK1/2 induced by IL‐1β at ATDC5 increased anabolism and decreased catabolism and apoptosis, while inhibiting CARM1 expression can inhibit the expression of p‐ERK1/2. Thus, CARM1 can modulate ATDC5 anabolism, catabolism, and apoptosis by phosphorylating ERK1/2. In addition, the overexpression of CARM1 at ATDC5 induced by IL‐1β promoted the expression of p‐ERK1/2, decreased anabolism, and increased catabolism and apoptosis. However, inhibition of p‐ERK1/2 attenuated the effects of CARM1 overexpression‐induced decreased anabolism, increased catabolism, and increased apoptosis, and these results further suggest that CARM1 regulates OA‐associated degradation in IL‐1β‐treated ATDC5 through interaction and phosphorylation of ERK1/2.

Furthermore, although we demonstrated that CARM1 expression is upregulated during OA progression, the precise mechanisms driving this upregulation remain to be fully elucidated. Inflammatory cytokines like IL‐1β and TNF‐α, prevalent in OA, may upregulate CARM1 via the NF‐κB pathway. CARM1 acts as an NF‐κB coactivator, and our data showing increased CARM1 in IL‐1β‐treated chondrocytes suggest NF‐κB might enhance CARM1 expression to support inflammatory responses (Covic et al. 2005). Mechanical stress, another OA hallmark, activates ERK1/2, potentially increasing CARM1 expression through transcription factors like AP‐1, forming a feedback loop that amplifies cartilage damage, consistent with stress‐induced MAPK activation (Zou et al. 2002). Additionally, aging, a key OA risk factor, correlates with higher CARM1 levels in our aging mice and OA patients. Epigenetic changes, such as DNA methylation shifts, may enhance CARM1 promoter activity during aging (López‐Otín et al. 2013). Taken together, these findings suggest that inflammatory, aging, and oxidative stress cues in the OA microenvironment likely contribute to CARM1 upregulation and OA progression through the ERK1/2 signaling axis. However, these hypotheses align with existing literature but require further validation.

The limitations of this study include the following: Firstly, although our findings indicate an upregulation of CARM1 in mouse chondrocytes stimulated by degenerative cartilage tissue and the pro‐inflammatory cytokine IL‐1β in human OA patients, further research is required to elucidate the specific transcription factors responsible for the IL‐1β‐mediated upregulation of CARM1. Secondly, previous studies have shown a correlation between the development of histologic OA in mice following DMM and poor functional outcomes, including pain experience (Huesa et al. 2016). Our study suggests that the CARM1‐ERK1/2 axis contributes to cartilage and joint degeneration following DMM, aligning with previous research (Huesa et al. 2016). These pathological changes are likely to be associated with pain sensation and impaired joint function. Therefore, future investigations are warranted to explore the role of CARM1 in OA‐related pain and joint dysfunction.

## Author Contributions

J.Y. and J.T. contributed equally to this work. Y.W. and B.Z. were involved in the conceptualization and writing – review and editing. J.Y. and J.T. conducted data curation and writing – original draft. R.L. and X.Q. carried out formal analysis, methodology, and visualization. D.H., G.H., and T.Z. handled validation and visualization. J.Y., P.L., and Y.W. were responsible for funding acquisition and supervision. All authors approved the final manuscript.

## Disclosure

The funders had no role in study design, data collection, interpretation and analysis, decision to publish, or preparation of the manuscript.

## Ethics Statement

This study obtained ethical approval from the Ethics Committee of the Second Hospital of Shanxi Medical University (approval number: 2021YX Number 019), and written informed consent was obtained from all participants. The experimental protocols involving C57BL/6 mice were conducted in strict accordance with the guidelines provided by the Ethics Committee of the Animal Transformation Center of Shanxi Medical University (approval number: DW2023013).

## Conflicts of Interest

The authors declare no conflicts of interest.

## Supporting information


Data S1.

## Data Availability

The data involved in this study are available from the corresponding author upon reasonable request.
