# Supplementary material for: Coactivator Associated Arginine Methyltransferase 1 Modulates Cartilage Degeneration and Chondrocyte Apoptosis in Osteoarthritis by Regulating ERK1/2 Signaling Pathway
Source: Aging Cell. 2025 Jul 3;24(8):e70122. doi: 10.1111/acel.70122 (PMC12341778; doi:10.1111/acel.70122)

**Supplementary Table1：Primer sequences used in this study**

| **Gene** | **Forward(5ʹ-3ʹ)** | **Reverse(5ʹ-3ʹ)** |
| --- | --- | --- |
| GAPDH | ACCCAGAAGACTGTGGATGG | CACATTGGGGGTAGGAACAC |
| Caspase3 | AGCTTGGAACGGTACGCTAA | GAGTCCACTGACTTGCTCCC |
| MMP13 | ACCTATTCCTGGTTGCTGCTC | AAACGGGACAAGTCTGTGGAG |
| ACAN | CCAAACCAGCCTGACAACTT | tctagcatgctccaccactg |
| CARM1 | ATCAGGAGATTACACTGACTGGA | GAGCATCCTCTTGCGTGGATT |
| COL2A1 | gccaagacctgaactctgc | gccatagctgaagtggaagc |

**Supplementary Table2：Mass spectrometry results of immunoprecipitation**

| Gene names | Unique peptides | Sequence coverage [%] | Mol. weight [kDa] | LV NC-1 | LV NC-2 | LV NC-3 | LV CARM1-1 | LV CARM1-2 | LV CARM1-3 |
| --- | --- | --- | --- | --- | --- | --- | --- | --- | --- |
| S100a10 | 2 | 27.8 | 11.186 | NaN | NaN | NaN | 494980000 | 537170000 | 464340000 |
| Tpm3 | 2 | 13 | 32.994 | NaN | NaN | NaN | 129288500 | 91157000 | 167420000 |
| Anxa11 | 2 | 5 | 54.079 | NaN | NaN | NaN | 106952000 | 85424000 | 128480000 |
| Esyt1 | 2 | 2.2 | 121.55 | NaN | NaN | NaN | 40602000 | 54870000 | 47736000 |
| Cdk13;Cdk12 | 2 | 3.2 | 164.55 | NaN | NaN | NaN | 26202000 | 81276000 | 136350000 |
| Poldip3 | 4 | 12.6 | 46.132 | NaN | NaN | NaN | 47026500 | 33056000 | 60997000 |
| Gltscr2 | 3 | 8.3 | 55.792 | NaN | NaN | NaN | 58478000 | 64269000 | 112750000 |
| Mepce | 3 | 5.4 | 72.05 | NaN | NaN | NaN | 90636000 | 70484000 | 80560000 |
| Cspg4 | 8 | 4.8 | 252.31 | NaN | NaN | NaN | 99291000 | 88192000 | 110390000 |
| Sfxn3 | 2 | 8.7 | 35.406 | NaN | NaN | NaN | 68860000 | 61170000 | 53480000 |
| Ubap2 | 2 | 2.7 | 117.96 | NaN | NaN | NaN | 64166000 | 58523000 | 52880000 |
| Trim26 | 2 | 6.2 | 62.808 | NaN | NaN | NaN | 67817000 | 69790000 | 35464000 |
| Mrps16 | 3 | 26.7 | 15.192 | NaN | NaN | NaN | 850190000 | 935390000 | 819860000 |
| Mapk1 | 2 | 17.8 | 41.275 | NaN | NaN | NaN | 47810000 | 72180000 | 56912000 |
| Mapk3 | 2 | 16.1 | 43.066 | NaN | NaN | NaN | 39427000 | 62450000 | 49457000 |
| Gmfb | 2 | 23.9 | 16.723 | NaN | NaN | NaN | 35204000 | NaN | 41153000 |
| Rbm7 | 2 | 7.2 | 30.148 | NaN | NaN | NaN | 67353000 | 65349000 | NaN |
| Ddx28 | 2 | 4.6 | 59.514 | NaN | NaN | NaN | NaN | 56572000 | 42233000 |
| Lima1 | 2 | 3.3 | 84.059 | NaN | NaN | NaN | 48351000 | NaN | 51487000 |
| Twf2 | 2 | 10.3 | 39.47 | NaN | NaN | NaN | NaN | 54629000 | 43486000 |
| Carm1 | 9 | 22.2 | 65.853 | NaN | NaN | NaN | 1386600000 | 675370000 | 1925200000 |

**Note:** NaN represents that the protein was not detected.

**Supplementary Figures**

**Supplementary Figure 1. Volcano plot of CARM1 overexpression in degenerative cartilage.** (a) The expression of CARM1 in the GSE103416 dataset displayed through bioinformatics analysis..


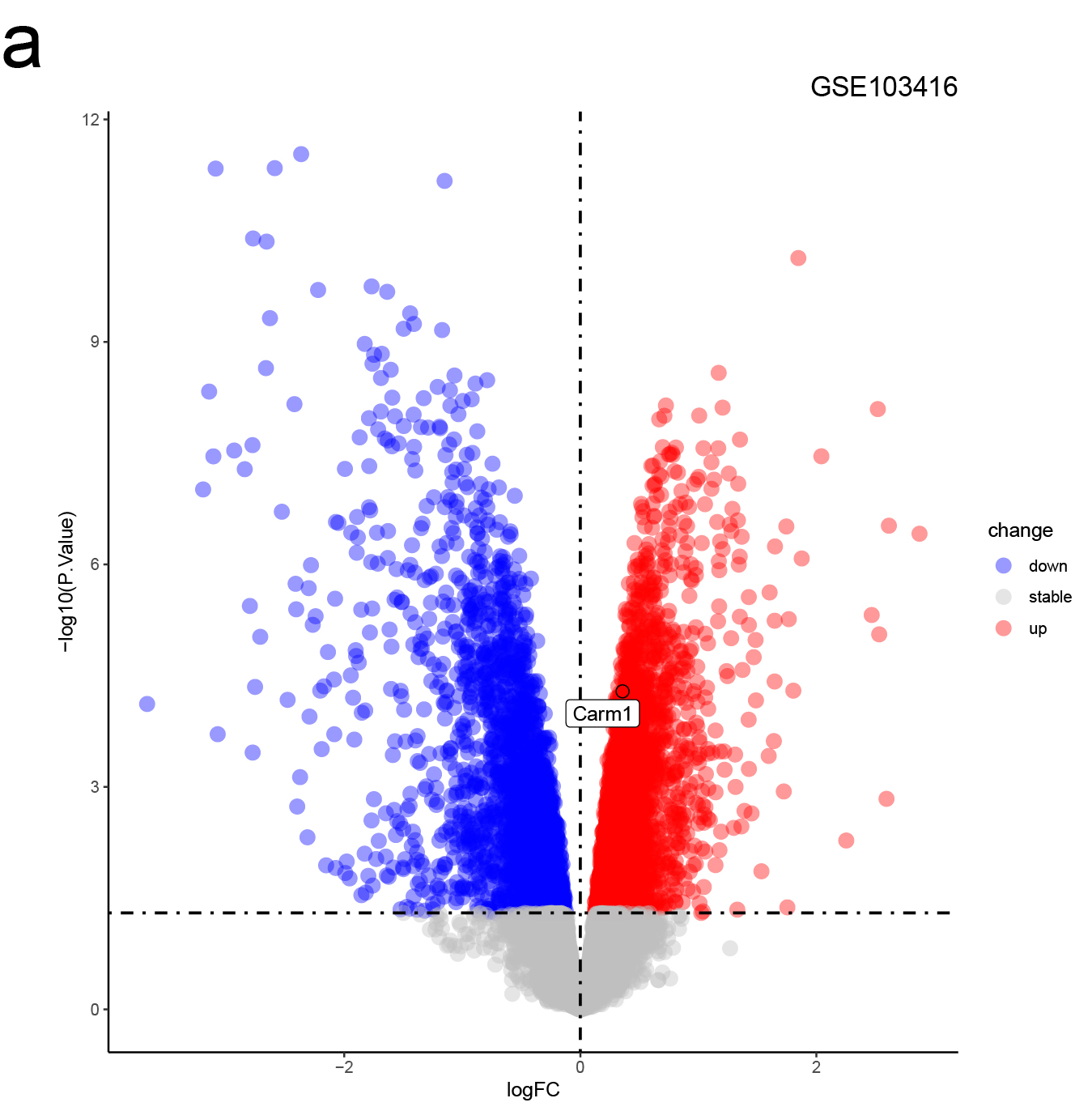


**Supplementary Figure 2. Isolation and culture of C57BL/6 mouse articular chondrocytes.** (a) Schematic diagram of isolation and culture of C57BL/6 mouse articular chondrocytes. (b) Representative images of isolated chondrocytes. (c) Identification of mouse chondrocytes using Safranin O, Alcian Blue, and IHC staining of COL2A1.


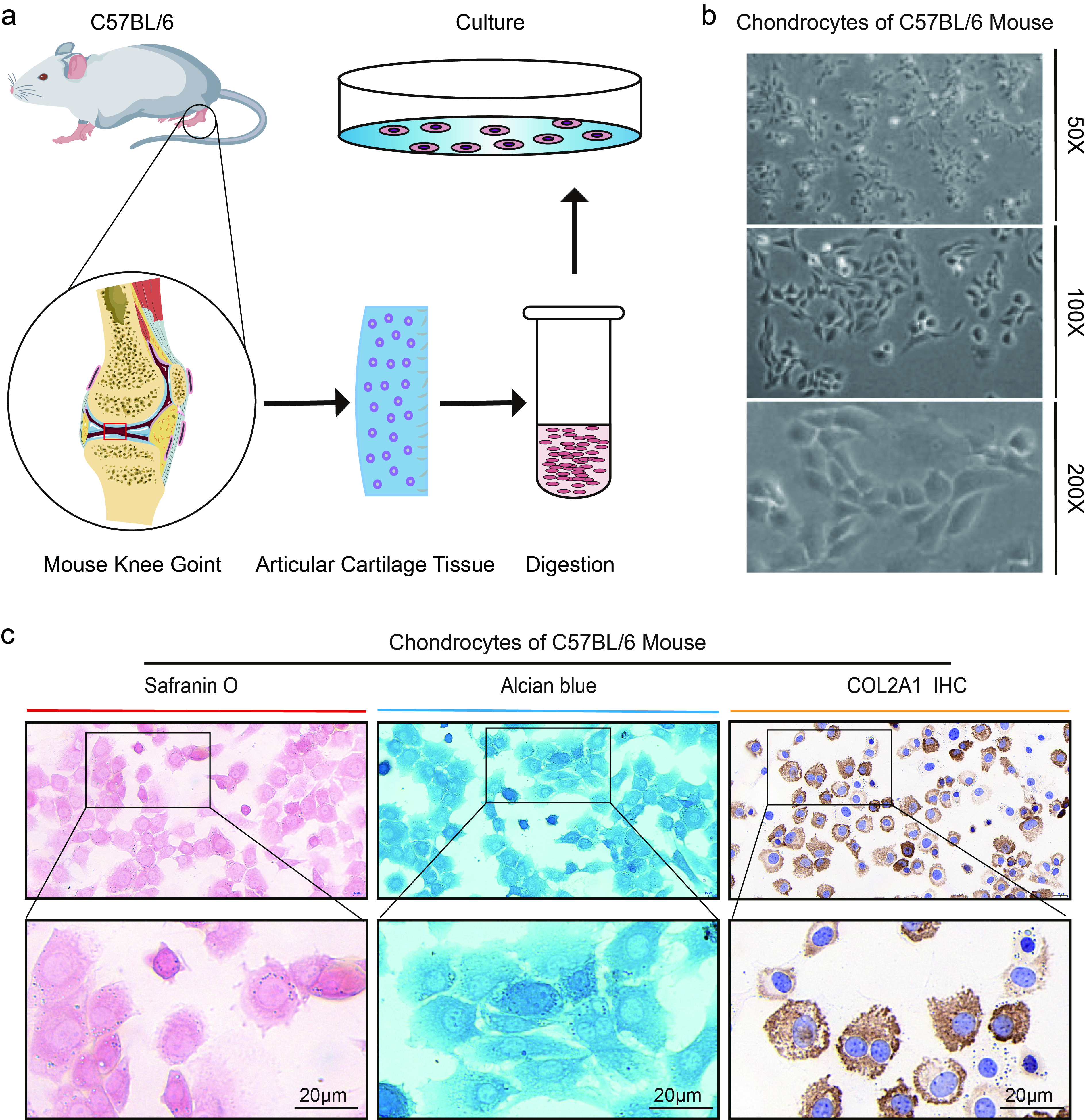


**Supplementary Figure 3. CARM1 is up-regulated in IL-1β-induced mouse chondrocytes.** (a,b) Western blotting and quantification shows the levels of CARM1, MMP13, and COL2A1 proteins in IL-1β-treated (0ng/ml, 5ng/ml, 10ng/ml, 20ng/ml) mouse chondrocytes. (c,d) RT-qPCR analysis shows the levels of CARM1, MMP13, and COL2A1 proteins in IL-1β-treated (0ng/ml, 5ng/ml, 10ng/ml, 20ng/ml) mouse chondrocytes. (e) CARM1 immunofluorescent staining of mouse chondrocytes treated with IL-1β (20ng/ml) for 12 h. (F) Quantitative results of CARM1 immunofluorescence staining. Data are presented as the mean ± SD; *p < 0.05, **p < 0.01, ***p < 0.001.


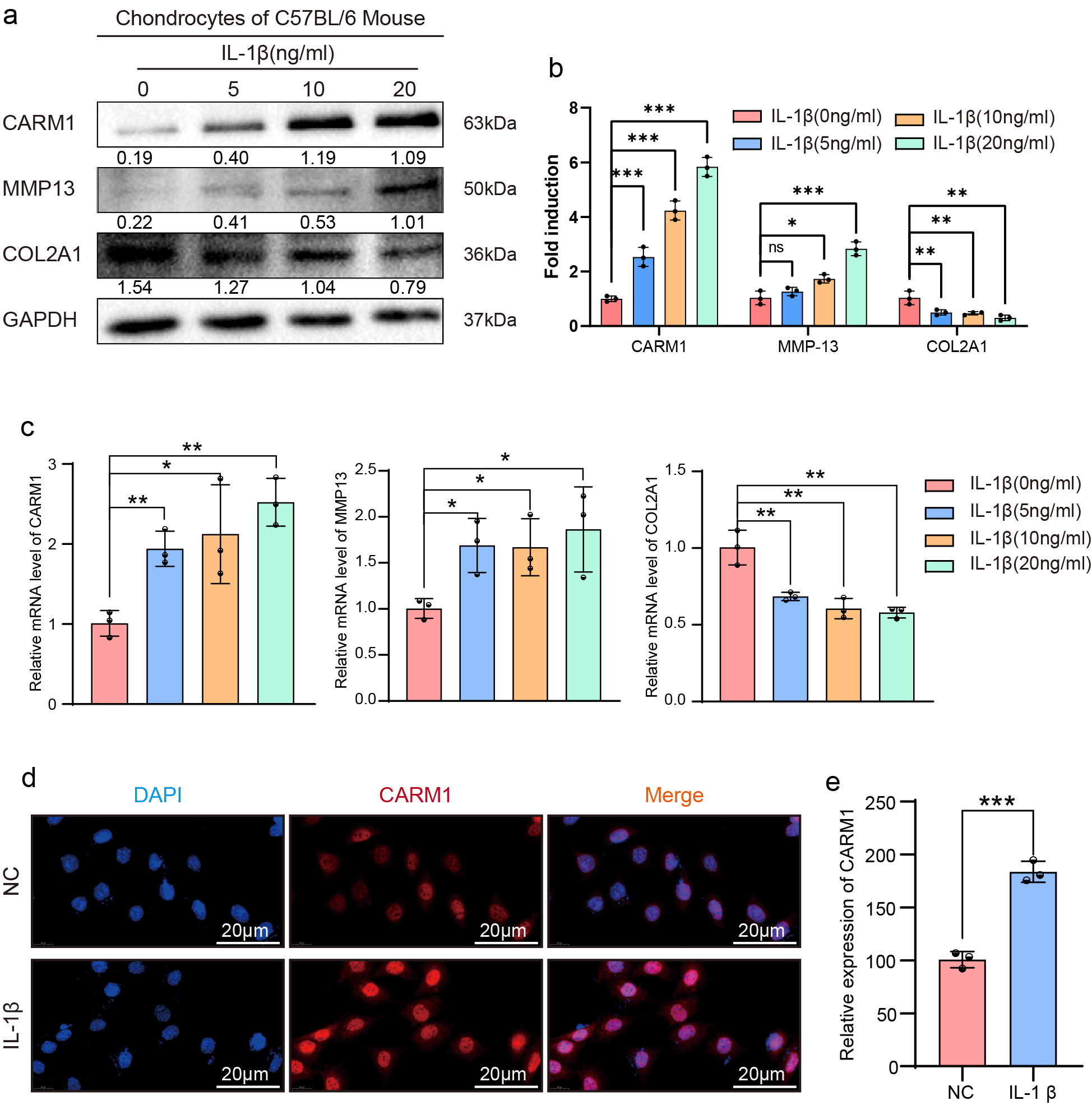


**Supplementary Figure 4. CARM1 inhibitor inhibited the CARM1 release in IL-1β-induced ATDC5.** (a) The structure of CARM1 inhibitor. (b) Viability of ATDC5 cultured with 0-40ng/ml of CARM1 inhibitor for 12 h. (c) Viability of ATDC5 cultured with 0-40ng/ml of CARM1 inhibitor for 24 h. (d) Viability of ATDC5 cultured with 0-40ng/ml of CARM1 inhibitor for 48 h. Data are presented as the mean ± SD; *p < 0.05, **p < 0.01, ***p < 0.001.


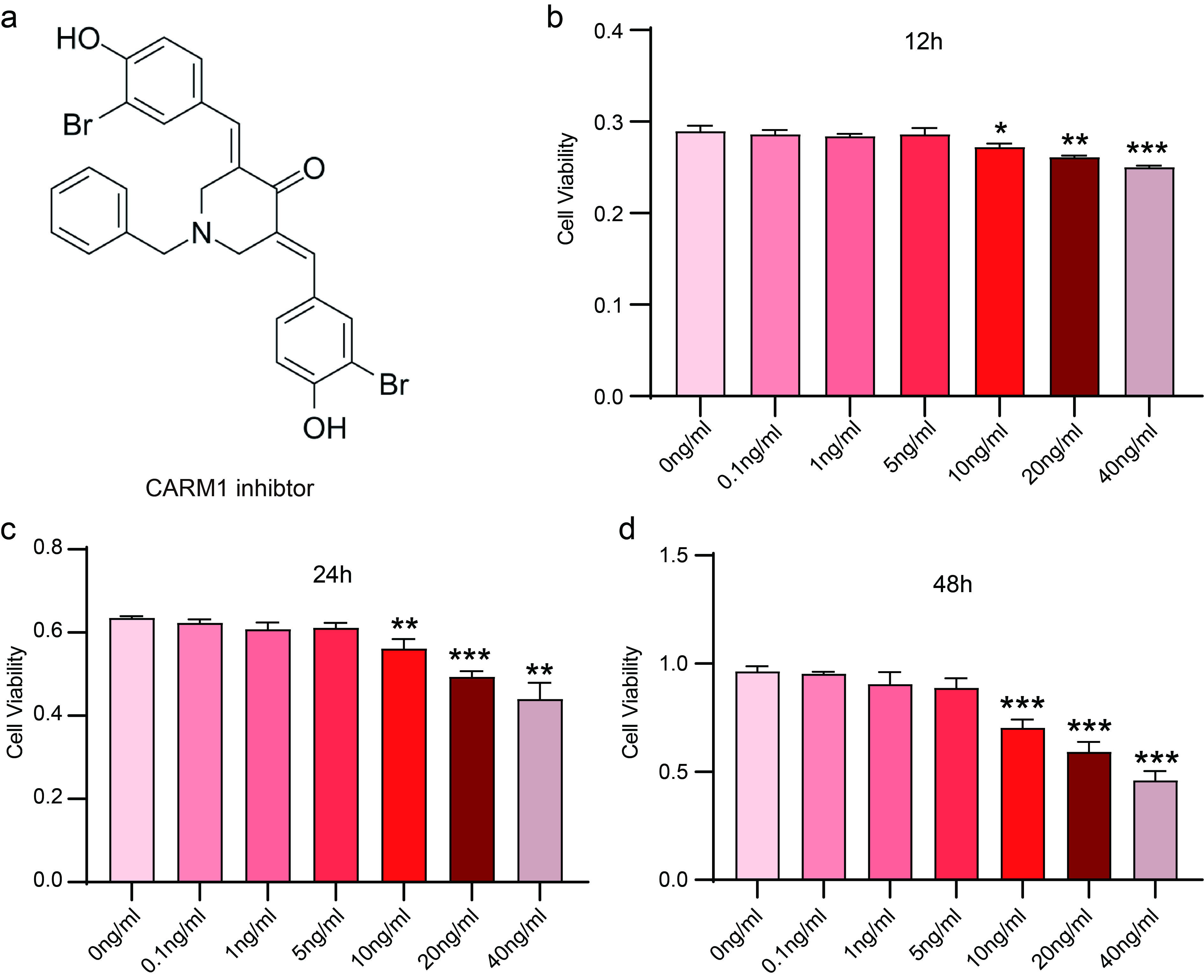


**Supplementary Figure 5. Inhibitor of CARM1 saved the decrease of ATDC5 proliferation induced by IL-1β.** (a) Representative images show EdU staining assay in ATDC5 treated with IL-1β or co-treatment with IL-1β and CARM1 inhibitor. (b) Quantitative analysis shows the total numbers of EdU positive in ATDC5 treated with IL-1β or co-treatment with IL-1β and CARM1 inhibitor. Data are presented as the mean ± SD; *p < 0.05, **p < 0.01, ***p < 0.001.


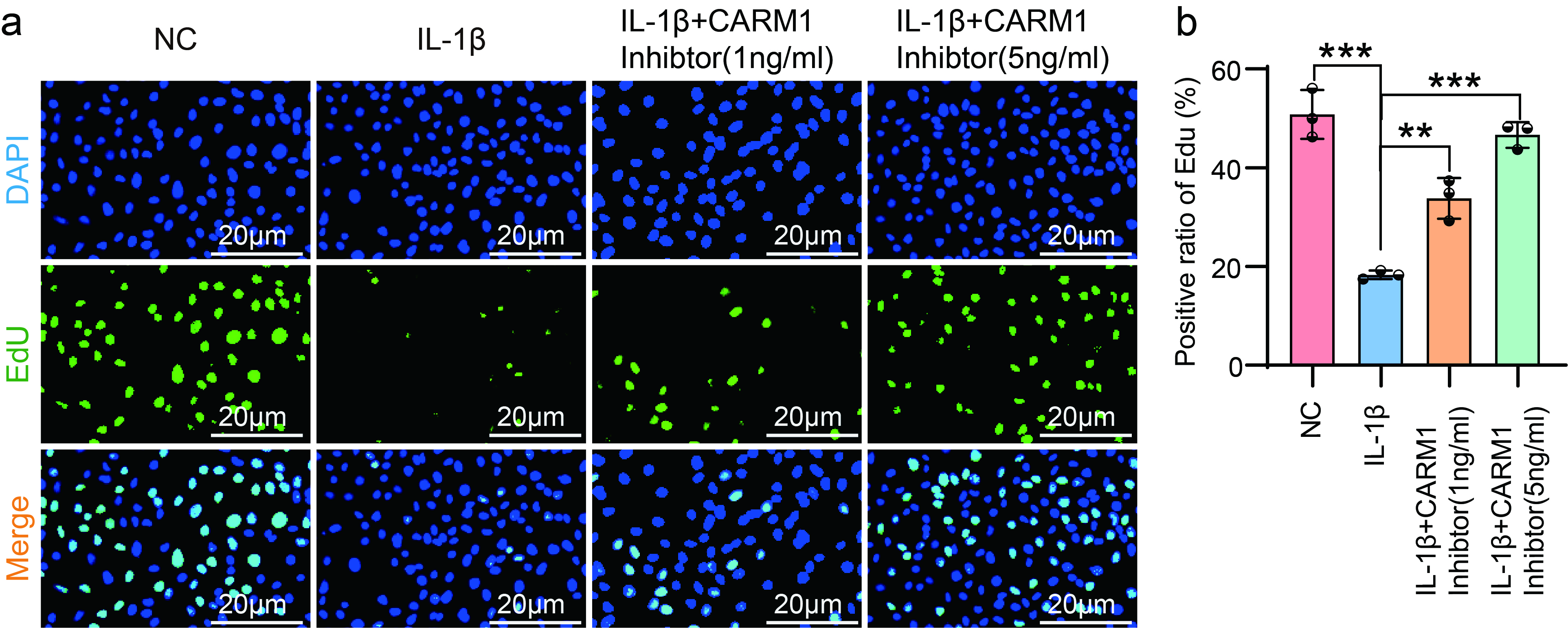


**Supplementary Figure 6. Inhibition of CARM1 rescued the degeneration of cartilage explant OA-related induced by IL-1β.** (a) Schematic diagram of in vitro culture of human articular cartilage explants. (b) Proteoglycan content was detected by Safranin O-Fast Green and Alcian Blue staining in cartilage explants after 2 weeks of incubation with IL-1β (20ng/ml) or CARM1 inhibitor (5ng/ml). (c) IHC staining of CARM1, ACAN and MMP13 in cartilage explants after 2 weeks of incubation with IL-1β (20ng/ml) or CARM1 inhibitor (5ng/ml). (d-f) Statistical analysis of the percentage of CARM1^+^, ACAN^+^, and MMP13^+^ chondrocytes by IHC staining. Data are presented as the mean ± SD; *p < 0.05, **p < 0.01, ***p < 0.001.


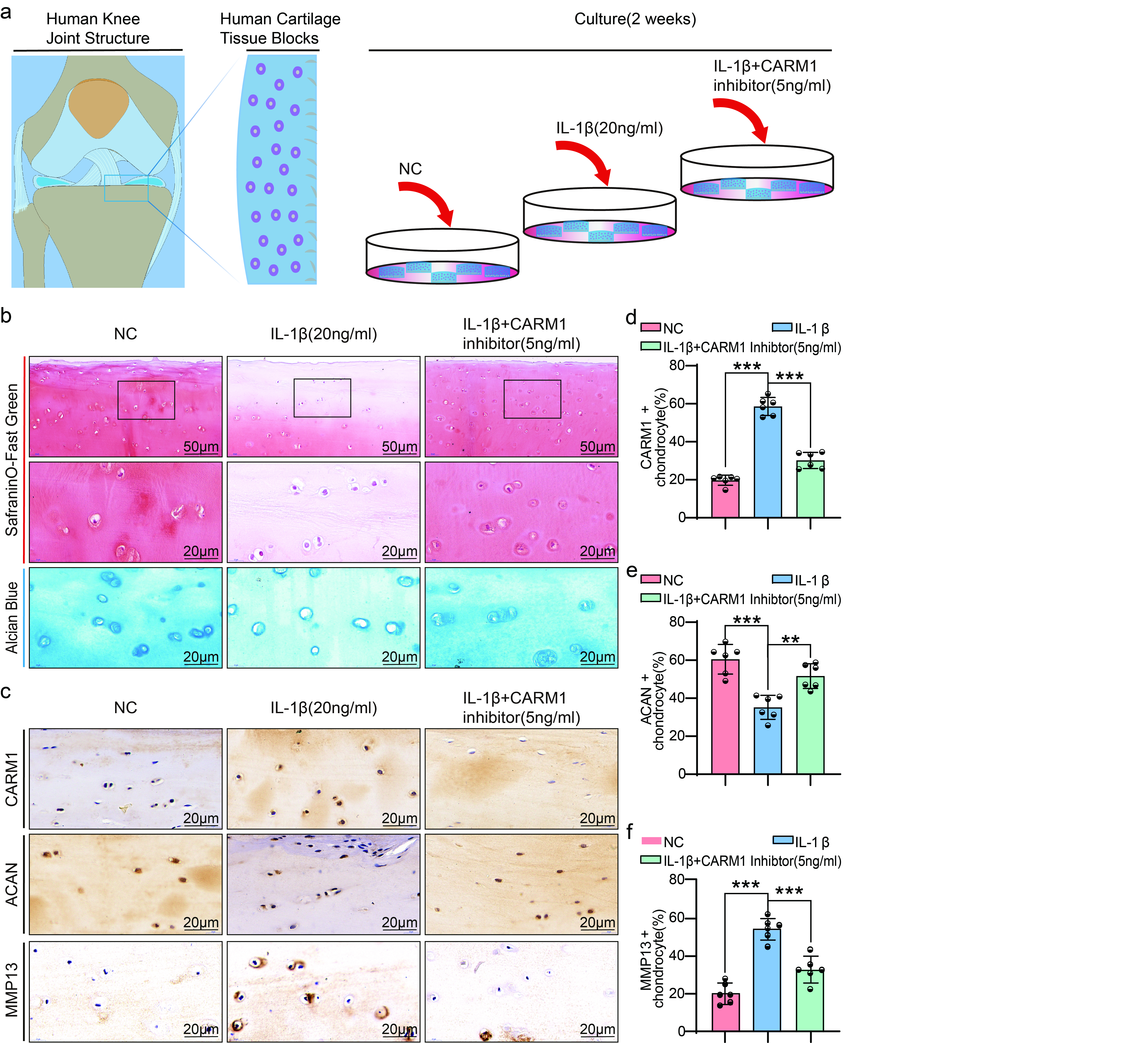


**Supplementary Figure 7. Over-expression of CARM1 inhibited the proliferation of ATDC5.** (a) Representative images show EdU staining assay in ATDC5 transfected with CARM1 over-expression lentivirus. (b) Quantitative analysis shows the total numbers of EdU positive in ATDC5 transfected with CARM1 over-expression lentivirus. (c) CCK-8 activity assay in ATDC5 transfected with CARM1 over-expression lentivirus. Data are presented as the mean ± SD; *p < 0.05, **p < 0.01, ***p < 0.001.


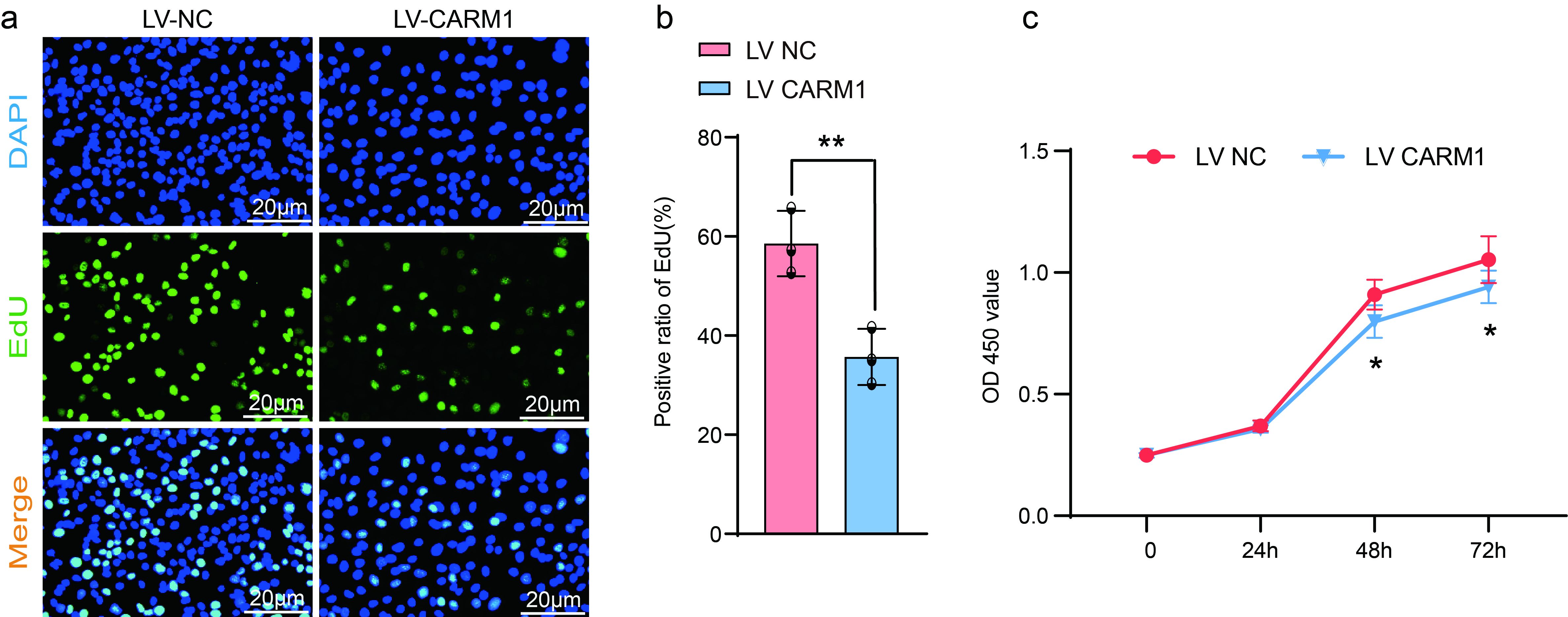


**Supplementary Figure 8. Over-expression of CARM1 exacerbated OA-related degeneration in DMM-induced mice.** (a) Representative images of plain radiographs from Sham, DMM, DMM+LV-NC, DMM+LV-CARM1, and DMM+LV-CARM1+CARM1 inhibitor groups (n=6 per group). (b) Quantitative analysis shows the articular space width from Sham, DMM, DMM+LV-NC, DMM+LV-CARM1, and DMM+LV-CARM1+CARM1 inhibitor groups (n=6 per group). Data are presented as the mean ± SD; ns: not significant, *p < 0.05, **p < 0.01, ***p < 0.001.


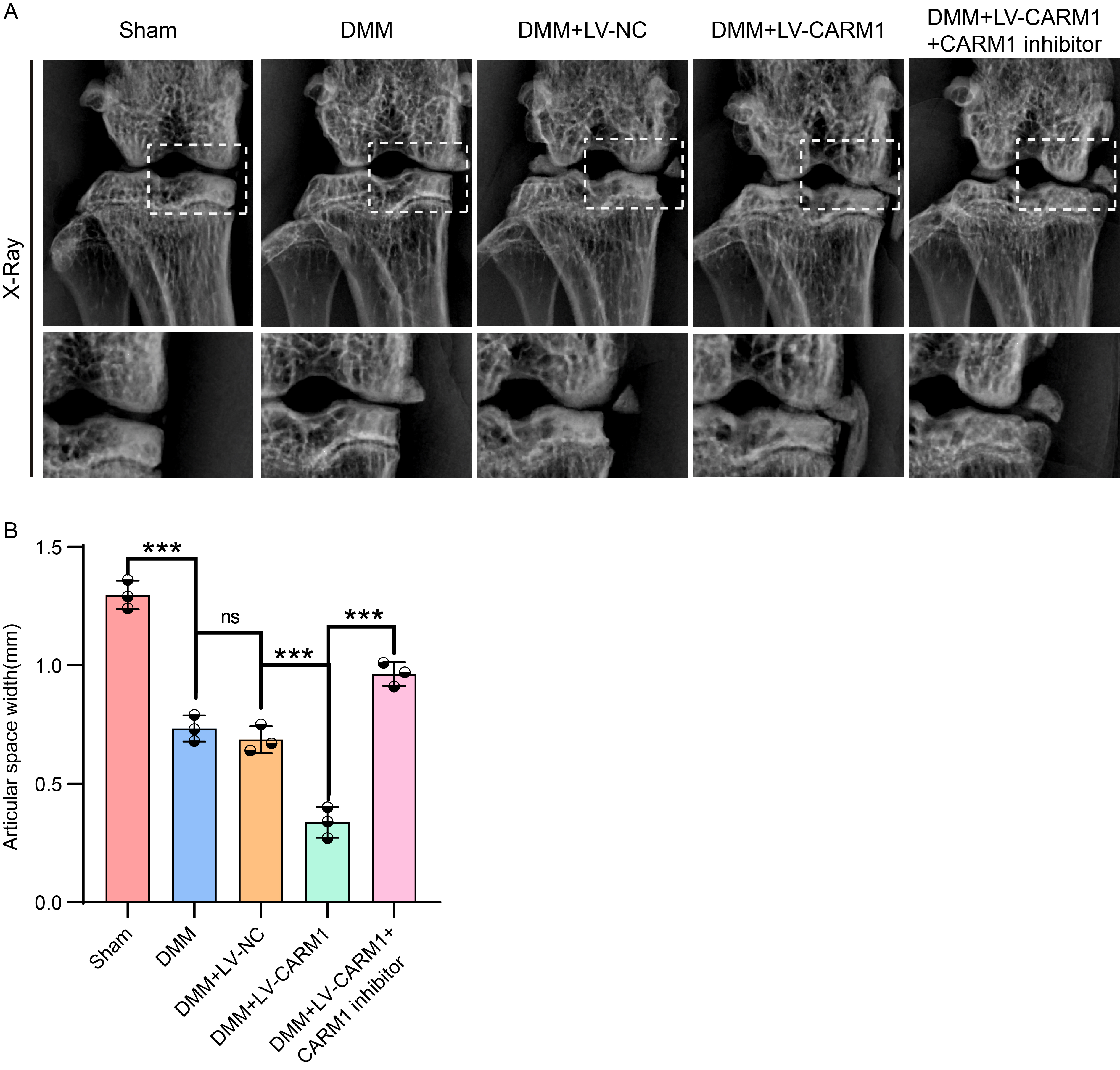


**Supplementary Figure 9. Protein-protein interaction (PPI) analysis of the proteins after CoIP.** (a) Protein-protein interaction analysis of the proteins precipitated with CARM1 using the STRING database.


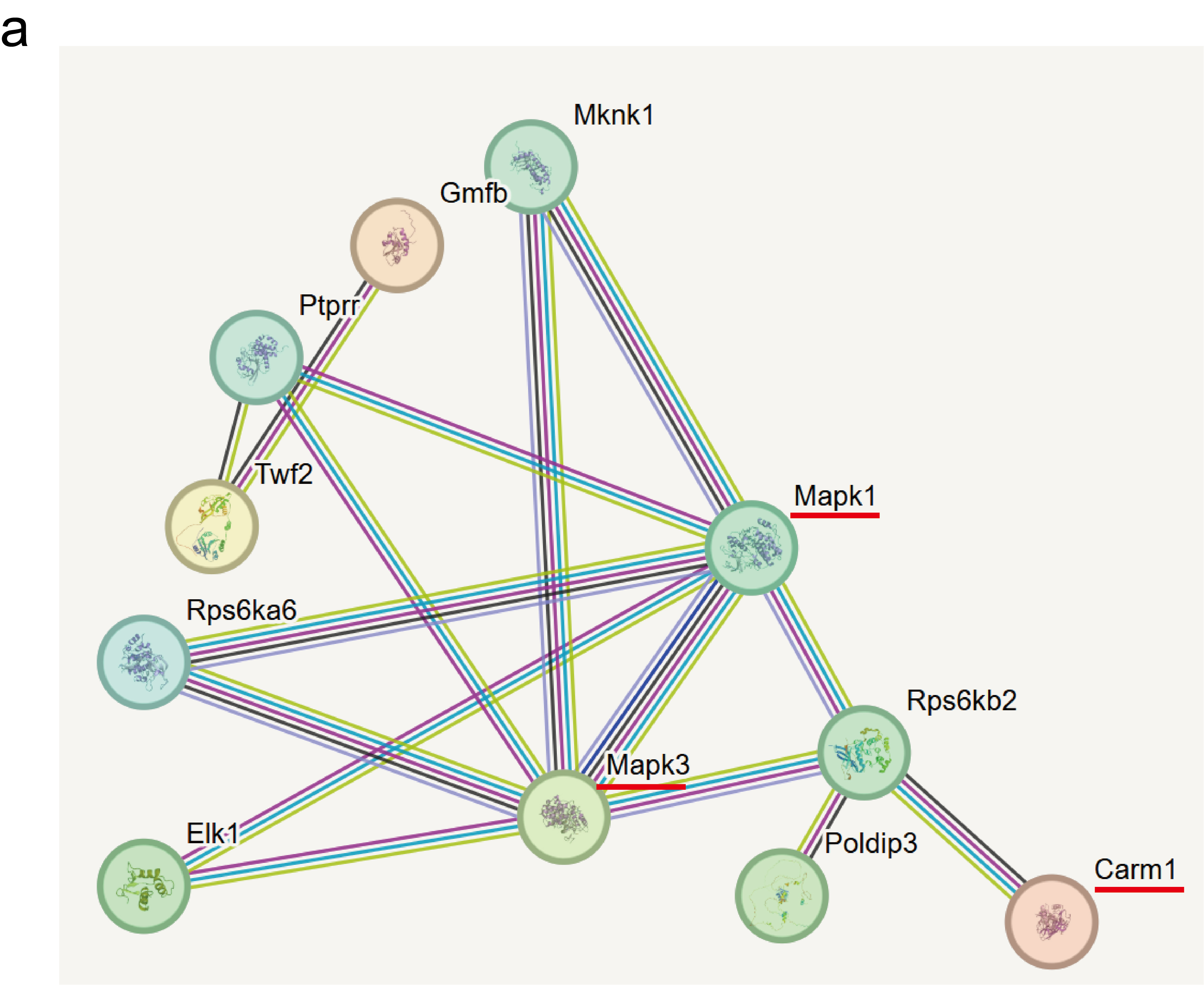


**Supplementary Figure 10. The impact of aging and inflammatory factor stimulation on the ERK1/2 pathway in chondrocytes.** (a,b) Western blotting and quantification shows the protein levels of P16, P21, ERK1/2,and p-ERK1/2 in P0 and P4, with GAPDH as the endogenous control. (c,d) Western blotting and quantification of ERK1/2 and p-ERK1/2 in normal and IL-6–stimulated ATDC5, with GAPDH as the endogenous control. (e,f) Western blotting and quantification of ERK1/2 and p-ERK1/2 in normal and TNF-α–stimulated ATDC5, with GAPDH as the endogenous control. Data are presented as the mean ± SD; *p < 0.05, **p < 0.01, ***p < 0.001.


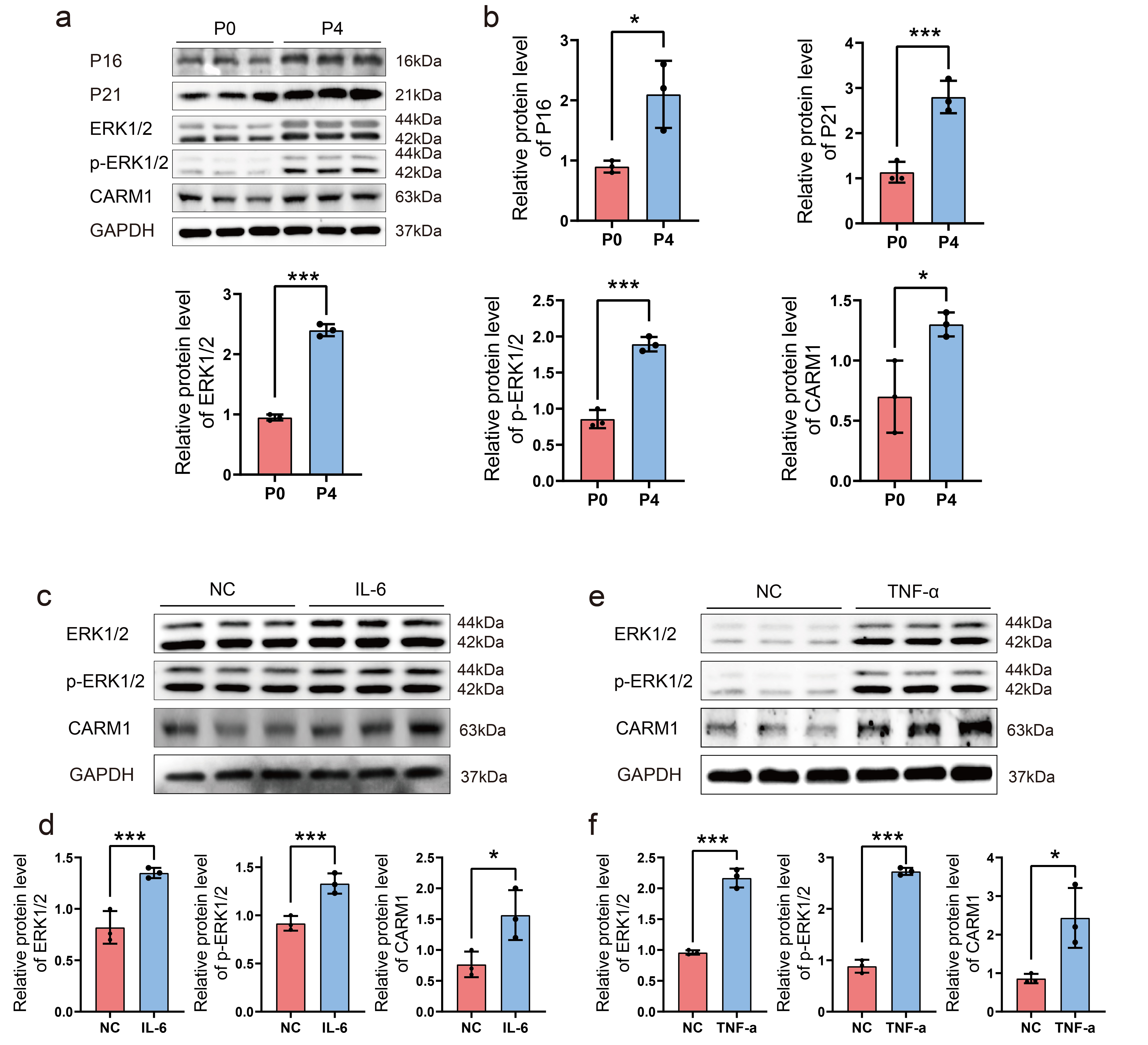


**Supplementary Figure 11. The effect of CARM1 modulation on the MAPK pathway.** (a,b) Western blotting and quantification shows the protein levels of CARM1, ERK1/2, p-ERK1/2, JNK, p-JNK, P38and p-P38 in the control and overexpression groups, with GAPDH as the endogenous control. (c,d) Western blotting and quantification shows the protein levels of CARM1, ERK1/2, p-ERK1/2, JNK, p-JNK, P38and p-P38 in the control and inhibitor groups, with GAPDH as the endogenous control. Data are presented as the mean ± SD; ns: not significant, *p < 0.05, **p < 0.01, ***p < 0.001.


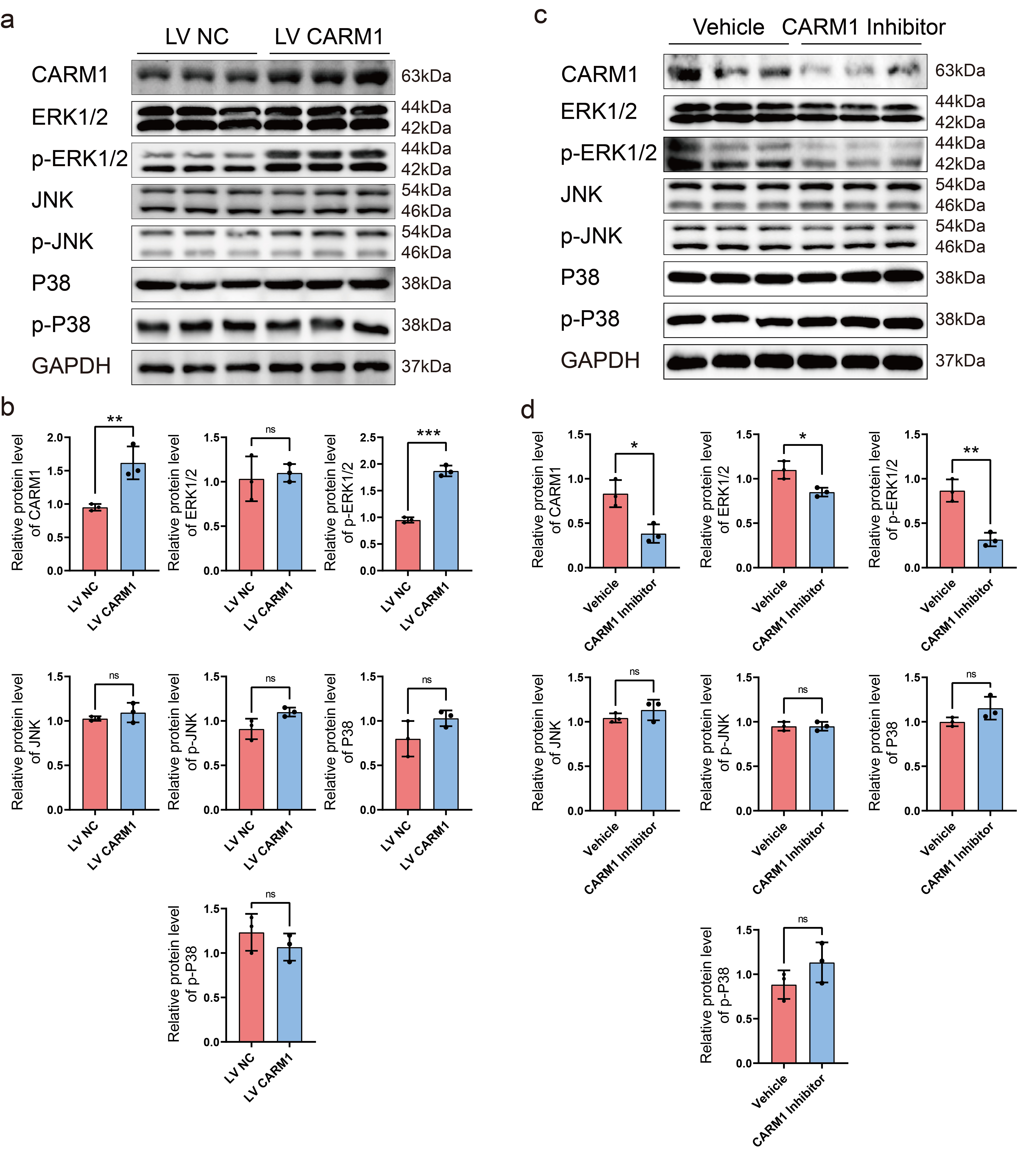

Supplement: Supplementary file 1 — Data S1. [file ACEL-24-e70122-s001.docx]
